# Role of Virus-Induced Host Cell Epigenetic Changes in Cancer

**DOI:** 10.3390/ijms22158346

**Published:** 2021-08-03

**Authors:** Valeria Pietropaolo, Carla Prezioso, Ugo Moens

**Affiliations:** 1 Department of Public Health and Infectious Diseases, “Sapienza” University, 00185 Rome, Italy; carla.prezioso@uniroma1.it; 2 IRCSS San Raffaele Roma, Microbiology of Chronic Neuro-Degenerative Pathologies, 00161 Rome, Italy; 3 Molecular Inflammation Research Group, Department of Medical Biology, Faculty of Health Sciences, University of Tromsø—The Arctic University of Norway, 9037 Tromsø, Norway

**Keywords:** chromatin remodeling, circular RNA, DNA methylation, histone modification, non-coding RNA, oncogenes, tumor suppressor genes, tumor virus

## Abstract

The tumor viruses human T-lymphotropic virus 1 (HTLV-1), hepatitis C virus (HCV), Merkel cell polyomavirus (MCPyV), high-risk human papillomaviruses (HR-HPVs), Epstein-Barr virus (EBV), Kaposi’s sarcoma-associated herpes virus (KSHV) and hepatitis B virus (HBV) account for approximately 15% of all human cancers. Although the oncoproteins of these tumor viruses display no sequence similarity to one another, they use the same mechanisms to convey cancer hallmarks on the infected cell. Perturbed gene expression is one of the underlying mechanisms to induce cancer hallmarks. Epigenetic processes, including DNA methylation, histone modification and chromatin remodeling, microRNA, long noncoding RNA, and circular RNA affect gene expression without introducing changes in the DNA sequence. Increasing evidence demonstrates that oncoviruses cause epigenetic modifications, which play a pivotal role in carcinogenesis. In this review, recent advances in the role of host cell epigenetic changes in virus-induced cancers are summarized.

## 1. Introduction

Viruses are infectious agents that can cause malignant and non-malignant diseases. Approximately 15% of all human cancers have a viral etiology and six human viruses are firmly associated with cancer [1]. They include the RNA viruses human T-lymphotropic virus 1 (HTLV-1) and hepatitis C virus (HCV), and the DNA viruses Merkel cell polyomavirus (MCPyV), high-risk human papillomaviruses (HR-HPVs), Epstein-Barr virus or human herpes virus-4 (EBV/HHV-4), Kaposi’s sarcoma-associated herpes virus or human herpesvirus-8 (KSHV/HHV-8) and hepatitis B virus (HBV) [2,3,4]. Despite their differences in structure and genome, all human tumor viruses apply the same mechanisms to induce oncogenesis. They convey the hallmarks of cancer on the host cell. Human viral oncoproteins will cause cells to evade growth suppression and apoptosis, to sustain proliferation and immortalization, to induce mutations and genome instability, to promote chronic inflammation, invasion/metastasis and angiogenesis, to escape immune destruction, and to deregulate cellular energetics [5,6]. Many of these processes are brought about by virus-mediated changes in gene expression because viral oncoproteins can directly modulate gene expression by activating transcription factors, inhibiting transcriptional repressors, and acting as transcription factors [5,6]. Oncoviruses can also affect cellular gene expression by epigenetic mechanisms, including modifying host DNA methylation, inducing chromatin remodeling, expressing viral-encoded non-coding RNAs such as microRNAs, long non-coding RNAs (lncRNAs) and circular RNAs (circRNAs), and changing cellular non-coding RNAomics [7].

It is very difficult to study the epigenetic changes in virus-induced cancer cells for several reasons. Tumors are usually not detected in an early stage and tumor cells represent end products rather than initiation products. Moreover, oncoviruses have often a very long incubation time and virus-induced tumors often occur several decades after the original infection [8,9,10]. It is challenging to differentiate between an epigenetic change that is directly due to viral infection, due to the host antiviral response or due to a subsequent downstream effect of the transformation process [11]. In vitro infection studies with human oncoviruses may give an idea of the initial epigenetic changes triggered by viral infection, but for oncoviruses such as HPV, MCPyV and HBV good cell systems are lacking.

Viruses also employ epigenetic changes to regulate their life cycle. This review focuses predominantly on the role of virus-induced epigenetic modifications of the host cell in carcinogenesis. The reader is referred to excellent reviews that expound how epigenetic changes modulate the viral life cycle replication [12,13,14,15].

## 2. Oncoviruses and Host Cell DNA Methylation

### 2.1. The Cellular DNA Methylation Machinery

DNA methylation occurs at cytosine residues in CpG dinucleotides and is a fundamental mechanism in silencing gene transcription and is catalyzed by a family of DNA methyltransferases (DNMTs). DNMT3A and DNMT3B are responsible for establishing DNA methylation. DNMT3L is catalytically inactive but stimulates the enzymatic activity of DNMT3A/3B. DNMT1 is responsible for maintaining the DNA methylation pattern. Erasing DNA methylation is executed by the demethylating enzymes ten-eleven translocation (TET), activation-induced cytidine deaminase (AICDA) and thymine DNA glycosylase (TDG). Methylation of DNA reduces gene expression, whereas demethylation has the opposite effect. Methylation of DNA can prevent transcription regulatory proteins to bind or allow proteins with high affinity for methylated CpG to bind. There are three families of such proteins: methyl-CpG-binding domain (MBD), ubiquitin-like, containing PHD and RING finger domain (UHRF), and Zinc-finger domain. The MBD family comprises MeCP2, MBD1, MBD2, MBD3, and MBD4. The UHRF family contains UHRF1 and UHRF2, and the last family includes Kaiso, Zinc finger and BTB domain containing 4 (ZBTB4) and ZBTB38 [16,17]. MeCP2 and MBD2 act as transcription repressors by recruiting histone deacetylases (HDACs), the nucleosome remodeling complex (NuRD), and the transcriptional repressor switch independent 3A (SIN3A) [18,19]. However, both MeCP2 and MBD2 were shown to function as transcriptional activators [20,21]. The other CpG binding proteins have been less studied.

Aberrant methylation is associated with diseases, including cancer [22,23]. Induction of de novo (de)methylation is one of the common mechanisms used by all human tumor viruses to alter host cell gene expression. Remarkably, virus-induced (de)methylation is non-random and occurs at CpG islands of specific genes, whose role in cancer has been well-established. This will be discussed for each human tumor virus in Section 2.2, Section 2.3, Section 2.4, Section 2.5, Section 2.6, Section 2.7 and Section 2.8 and the effects of viral oncoproteins on enzymes involved in CpG methylation are summarized in Table 1.

### 2.2. HTLV-1 and Host Cell DNA Methylation

The retrovirus HTLV-1 infects 10–20 million people worldwide, but only 3–5% of infected individuals will develop adult T-cell leukemia-lymphoma (ATL) 30–50 years after initial infection [57,58]. HTLV-1 is also linked to a neurodegenerative disease called tropical spasticparaparesis/HTLV-I–associated myelopathy [59]. The viral proteins Tax and basic zipper (HBZ) are crucial for tumorigenesis [60,61,62]. However, not all ATL tumor cells express Tax and during the late stage of leukemogenesis, Tax expression is frequently inactivated through several mechanisms such as loss of or DNA hypermethylation of the 5′ long terminal repeat (LTR) or nonsense, insertion or deletion mutations in the *Tax* gene, suggesting that the Tax protein is not essential for the maintenance of ATL [63]. HBZ is transcribed as an antisense transcript of the HTLV-1 provirus and is constitutively expressed in all ATL cases [64].

The integrated HTLV-1 genome is often hypermethylated. Tax was able to increase the transcriptional activity of HLTLV-1 LTR even when heavily methylated [25]. Stimulation of hypermethylated LTR by Tax required association with MDB2. Tax and MBD2 possibly target other methylated sequences and activate transcription from methylated promoters. Indeed, Tax:MBD2 could activate methylated cAMP-response element (CRE) containing promoters [25], suggesting that Tax may induce expression of cellular CRE containing promoters, even if they are hypermethylated. Genome-wide analysis has identified approximately 4000 CRE-containing promoters in the human genome [65], whose expression may be affected by Tax independently of their methylation state.

Methylation analysis of ATL genomes showed prominent CpG hypermethylation and hypomethylation in comparison with controls [66,67,68,69]. This altered methylation pattern was associated with transcriptional silencing and upregulation of cellular gene expression. Kruppel-like factor 4 (*KLF4*) and early growth response 3 (*EGR3*) were among the genes that were hypermethylated. Ectopic expression of KLF4 and EGR3 in ATL cell induced apoptosis, indicating that hypermethylated-mediated silencing of these genes enables ATL cell to escape from cell death [70]. Transcription factor-encoding genes and Major histocompatibility complex class I (*MHC-I*) genes were also hypermethylated. This may result in altered gene expression and may help ATL cells to evade the immune system [68,69]. Hypomethylated genes in ATL cells included PR/SET domain 16 (*PRDM16*), resulting in elevated expression of the protein encoded by the *PRSM16* gene, transcription factor *MEL1*. Overexpression of this protein is associated with leukemogenesis [67]. The *FOX3P* locus was found to be hypomethylated in cells from ATL patients and higher FOX3P protein levels were observed [71]. Tax was previously shown to reduce, whereas HBZ increased FOX3P expression [72,73]. However, Tax and HBZ levels did not relate to hypomethylation status of the FOX3P locus, suggesting that hypomethylation was not induced by HTLV-1 [71].

The mechanisms by which HTLV-1 enforces DNA methylation are incompletely understood. Although DNMT1 and DNMT3B were upregulated in HTLV-1 transformed T cells, not all cells expressed Tax, suggesting a Tax-independent mechanism [26]. The promoter of the tumor suppressor gene Src homology-2-containing protein tyrosine phosphatase (*SHP-1*) gene is hypomethylated in ATL cells and SHP-1 expression is lost. The authors showed that Tax repressed SHP-1 expression by recruiting HDAC1, but whether demethylation of the promoter depended on Tax was not investigated [24]. The tumor suppressor gene N-myc downregulated gene 2 (*NDRG2*) is frequently downregulated in ATL. Tax indirectly contributed to repression of this promoter by increasing the expression of enhancer of zeste homolog 2 (EZH2), a histone methyltransferase. Overexpression of EZH2 suppressed transcription of *NDRG2* via DNA methylation and trimethylation of histone 3 at lysine 27 (H3K27me3) [74]. Both examples suggest that Tax indirectly can modulate DNA methylation. Tax may induce irreversible changes in DNA methylation during the initial phase of HTLV-1 infection and this may explain why constitutive Tax expression is not required in ATL. Tax was shown to interact with coactivator associated arginine methyltransferase 1 (CARM1 or PRMT4), and this stimulated histone H3 methylation [75]. A possible role of HBZ in DNA methylation has not been divulged. Importantly, aberrant DNA methylation in ATL cells may not only be caused by HTLV-1 because aging and cancer are closely related to aberrant DNA methylation. The long incubation time of ATL and the prolonged life span of these cells might be predisposing factors for perturbed DNA methylation [76,77]. 

### 2.3. HCV and DNA Methylation

HCV is a (+) RNA virus belonging to the family Flaviviridae and is one of the leading causes of hepatocellular carcinoma (HCC). The viral genome is translated into a polypeptide of approximately 3000 amino acids that is cleaved by viral-encoded and cellular proteases to generate structural and non-structural proteins [78]. In vitro studies and transgenic animal models have shown that the viral proteins NS3, NS5A, and the core protein have oncogenic properties [6,78,79,80].

The methylation landscape of HCV-positive HCC tissues differs from non-tumor controls and a correlation between HCV infection and aberrant methylation of genes such as *CDKN2A* (cyclin-dependent kinase inhibitor 2A), *CDH1* (cadherin 1), *SOCS1* (suppressor of cytokine signaling 1), *RASSF1A* (Ras associated domain family member 1), APC (adenomatous polyposis coli protein), *GSTP1* (glutathione S-transferase Pi 1), *STAT1* (Signal transducer and activator of transcription 1), and *PRDM2* (PR/SET domain 2) in HCV-positive HCC has been established. Hampered expression of these genes contributes to cancer by promoting cell proliferation, mobility and invasion, and immune evasion [27,29,81,82,83,84]. The core protein seems to be implicated in HCV-induced DNA methylation because DNMT1 and DNMT3B levels were enhanced in HCV core protein expressing HepG2 cells and in Huh-7 cells compared to control cells [27,28,29,30]. The exact mechanisms by which the core protein induces expression of DNMT1 and DNMT3B is unknown but required activation of the STAT pathways by this viral protein [30]. Another possible mechanism, which is applied by the HBX protein of HBV (see Section 2.8), is through the retinoblastoma (pRb)/E2F pathway [53]. The *DNMT1* gene is an E2F1 target gene and the core protein has been shown to phosphorylate pRb, resulting in activation of E2F1-dependent transcription.

### 2.4. MCPyV and Host Cell DNA Methylation

MCPyV is the most recently identified virus to be linked to a human cancer. It is associated with about 80% of Merkel cell carcinoma (MCC), a rare, but aggressive cutaneous malignancy. The MCPyV genome is always integrated in all virus-positive MCCs examined [9,85]. MCPyV is a non-enveloped virus belonging to the *Polyomaviridae* family [86]. The viral oncoproteins are large tumor antigen (LT) and small tumor antigen (sT). In vitro and animal studies and the detection of sT in the absence of LT in some MCC indicate that sT may be more involved in the oncogenic process, whereas LT is required to sustain the tumor cell growth [85,87]. 

The DNA methylomes of MCPyV-negative and MCPyV-positve MCCs display significant differences in several genes that are associated with cancer. Frequent occurrence of *RASSF1A* promoter hypermethylation was observed in MCPyV-positive MCC [88]. DNA methylation examination of MCPyV-positive and MCPyV-negative MCC specimens showed that 54% had hypermethylation of the *RASSF1A* promoter and 22% of the *CDKN2A* promoter, whereas the promoters of the tumor suppressor genes fragile histidine triad diadenosine triphosphate (*FHIT*), tumor promoter p73 (*TP73*), and protein tyrosine phosphatase receptor type G (PTPRG) had no or infrequent hypermethylation. However, no significant correlation between viral infection and hypermethylation was observed, indicating that MCPyV infection may not induce DNA hypermethylation of these promoters [88]. Hypermethylation of the promoters of the *RASSF2*, *RASSF5A*, *RASSF5C* and *RASSF10* and the *TERT* gene (encoding telomerase reverse transcriptase) was frequently detected in MCCs compared to normal skin samples, but again no correlation with MCPyV infection was found [89,90]. The promoter of the *RB1* gene (encoding retinoblastoma protein pRb) was hypermethylated in MCCs compared to normal skin samples, but the pattern of hypermethylation of the RB1 promoter was similar in all MCCs independent of the MCPyV status [91]. MCPyV LT can inactivate pRb through interacting with the protein, suggesting the hypermethylation of the RB1 gene to inactivate expression is superfluous. However, the polyomavirus SV40 LT can both bind pRb and induced hypermethylation of the RB1 promoter in diffuse large B-cell type lymphomas [92]. This illustrates that LT of different polyomaviruses can possess distinct functions. The *INK4A-ARF* (*CDNK2A*) locus and *DUSP2* (dual specificity phosphatase 2) gene were found to be frequently hypermethylated in MCC tumors, but the viral status in these tumors was not specified, so that a possible role for MCPyV in hypermethylation cannot be determined [93,94]. In another study, no difference in *INK4A-ARF* methylation was found between virus-positive and virus-negative MCC tumors [95]. Hypomethylation of the *PTCH1* gene (encoding the Patched 1) and the gene for Atonal BHLH transcription factor 1 (*ATOH1*) was detected in both virus-negative and virus-positive MCC cell lines [96,97]. MCC is considered a neuroendocrine tumor and repressor element 1 silencing transcription factor (REST) is a key regulator in neuronal programs. Moreover, REST can act as an oncogene in neural cells and a tumor suppressor in non-neural cells. Therefore, Chteinberg et al. investigated the expression of REST in MCC. REST protein was not detected in any of the examined MCPyV-negative and MCPyV-positive tumors and MCPyV-negative and MCPyV-positive cell lines, but no hypermethylation of the *REST* promoter was observed in all tissues and cell lines, indicating that silencing of *REST* is not caused by hypermethylation and occurred independently of the virus status. The authors speculated that miR-9, which is upregulated in MCCs and targets the 3′ untranslated region of *REST* mRNA, may prevent REST synthesis [98]. The loss of O6-methylguanine-DNA methyltransferase expression has been associated with a wide variety of cancers. The O6-methylguanine-DNA methyltransferase promoter was hypermethylated in six MCPyV-positive MCC cell lines, but hypomethylated in 18 MCC tissues with unknown viral status [99]. This finding emphasizes that caution is warranted when comparing results from tumor cell lines and tumor tissue. 

In conclusion, aberrant DNA methylation of cancer-related genes is common in both MCPyV-negative and MCPyV-positive MCCs and does not seem to be provoked by MCPyV infection. Viral-independent modification of host DNA methylation was further confirmed in a study that showed that DNA methylation in MCC tissues was significantly lower as compared to the patients’ chronological age. The accelerated DNA methylation in patients was irrespective of the viral presence [100]. Although SV40 LT can upregulate the expression of DNMT3B, thereby contributing to the oncogenic phenotype in a lung cancer model [101], it is not recognized whether MCPyV LT can affect the expression levels or activity of specific DNMTs. A recent study demonstrated a correlation between MCPyV and the methylation pattern in MCC. The authors found that the programmed cell death 1 (*PDCD1*) promoter was hypomethylated in 42 out of 69 MCCs tissues and hypomethylation was significantly more frequent in virus-positive tumors. Virus-positive MCC patients with hypomethylated *PDCD1* promoter had a better prognosis than those with high *PDCD1* methylation [102]. Further studies are required to establish whether MCPyV infection has an effect on host DNA methylation.

### 2.5. High-Risk (HR) HPV and Host Cell DNA Methylation

Human papillomaviruses (HPV) are non-enveloped viruses with a circular dsDNA genome of approximately 8000 base-pairs [103]. More than 200 different types of HPV have been isolated and several of them, so called high risk HPV (HR-HPV) are associated with anogenital and oropharyngeal cancers [104]. HR-HPV are responsible for >99% of cervical cancer cases, with HPV16 (55% of all cases) and HPV18 (15% of all tumors) the two most common types [105]. In the USA about 40–80% of oropharyngeal cancers are positive for HR-HPV, whereas in Europe the incidence varies between 15% and 90%, with >90% of the cases containing HPV16 [106]. The main oncoproteins are E5, E6 and E7 (for a recent review see [107]).

Methylome analyses of HPV-positive cancers revealed differences in DNA methylation compared to matching normal tissue or HPV-negative tumors and transfection studies have confirmed that the E6 and E7 oncoproteins provoked hypermethylation tumor suppressor genes and hypomethylation of proto-oncogenes [31,32,108,109,110,111,112,113,114]. Both these viral proteins have been shown to upregulate the expression of DNMT1. E7 does so by derepressing E2F through sequesting pRb, whereas E6 inactivates p53, which abrogates the interaction of p53 with transcription factor Sp1 on the DNMT1 promoter. As the p53:Sp1 complex represses the *DNMT* promoter, E6 releases the repression by appropriating p53 [31,32]. Furthermore, E7 associates with DNMT1 and stimulates its activity [32]. Increased expression of DNMT3B was reported in non-smoking female lung cancer patients with HPV16 or HPV18 positive tumors, but the role of E6 and E7 was not investigated [115]. The mechanism(s) by which HR-HPV provoke hypomethylation of the host genome remain enigmatic. In conclusion, HPV-mediated changes in DNA methylation affects the expression of several cellular genes and has been proven to stimulate cell proliferation, cell survival, adhesion and migration [32,114].

### 2.6. EBV and Host Cell DNA Methylation

EBV or HHV4 is an enveloped virus with a dsDNA genome of around 170 kilobase-pairs. More than 90% of the world population have lifelong infection with this virus. EBV is associated with Burkitt’s lymphoma, Hodgkin’s disease, primary effusion lymphoma (PEL), nasopharyngeal carcinoma lymphoma, gastric carcinoma, but also with non-malignant diseases, including infectious mononucleosis [3,5,116]. EBV-induced cancer has an incidence of about 1 in 200,000 per year. The major EBV oncoprotein is LMP1, but other viral proteins including LMP2A, EBNA1, EBNA2, EBNA3 and EBNA-LP, and viral RNA transcripts (see further) are implicated in EBV-induced tumorigenesis [3,6,117].

EBV-associated cancers such as gastric cancer, nasopharyngeal carcinoma and Burkitt’s lymphoma are characterized by extensive hypermethylation of the host DNA compared with non-infected tumors and cell culture studies have illustrated that EBV infection induces de novo methylation [45,111,118,119,120,121,122]. Many of the genes whose expression is affected by EBV-induced methylation code for proteins involved in cell cycle control, signaling pathways, apoptosis, invasion and migration [45,111,122,123]. Some of these genes will be discussed, as well as the viral proteins involved in their methylation.

LMP1 induces hypermethylation of the *CDH1* promoter and downregulation of cadherin 1 by augmenting the expression and activity of DNMT1, 3A and 3B [33]. Loss of function of the *CDH1* gene contributes to cancer progression by increasing proliferation, invasion, and metastasis [124]. The gene for tumor suppressor *RASSF10*, which encodes a protein that inhibits cell proliferation, invasion, and migration and induces apoptosis was hypermethylated in EBV-positive gastric cancer compared to EBV-negative gastric cancers. The authors demonstrated that LMP1 promoted DNMT1 expression, which was responsible for hypermethylation of the *RASSF10* gene. Overexpression of LMP1 in human gastric adenocarcinoma AGS cells stimulated migration, invasion and cell colony formation and this was counteracted when RASSF10 was co-expressed. Xenograft studies with LMP1 and LMP1 plus RASSF10 cells confirmed that RASSF10 thwarted the LMP1-malignant phenotype. These results suggest that LMP1-mediated methylation and silencing of the *RASSF10* gene plays a role in EBV-induced oncogenesis [125]. Other studies confirmed that LMP1 upregulates DNMT1, DNMT3A and DNTM3B. LMP1-induces DNMT1 expression dependent on activation of the c-Jun N-terminal kinase (JNK)/AP1 pathway, whereas DNMT3A and DNMT3B were induced via the NFκB pathway [34,35]. LMP2A increased expression of DNMT1 via STAT3 and DNMT3A via the mitogen-activated protein kinase (MAPK) pathway and downregulated the expression of the demethylating enzymes TET1 and TET2 [39,40,41]. However, in germinal center B-cells, presumptive progenitors of Hodgkin’s lymphoma, EBV infection resulted in downregulation of DNMT1 and DNMT3B and upregulation of DNMT3A and the authors found that LMP1 is responsible for downregulation of DNMT1, while the mechanism for DNMT3A and DNMT3B remains unknown as ectopic expression of LMP1 or of LMP2A had no effect on DNMT3A and DNMT3B levels [38]. LMP2A caused hypermethylation of the phosphatase and tensin homolog (*PTEN*) gene through stimulation of DNMT1 in a STAT3-dependent manner [39]. EBNA3C, another EBV protein, could induce hypermethylation of the *RASSF1A* promoter by enhancing DNMT3A expression. This epigenetic modification results in decreased RASSF1A expression, leading to increased cell proliferation [42]. Finally, EBV-mediated methylation also affects genes whose products are involved in histone modification and chromatin remodeling. LMP1 could recruit DNMT1 to the promoter of the lysine-specific demethylase 2b (*KDM2B*) and trigger hypermethylation. KDM2B demethylates histone 3 at lysine 4 (H3K4me3). H3K4me3 is commonly associated with active transcription and demethylation will result in transcriptional silencing [37]. Thus, EBV-provoked changes in the host DNA methylation can contribute to virus-induced tumorigenesis.

### 2.7. KSHV and Host Cell DNA Methylation

KSHV or HHV8 is the causative agent of Kaposi sarcoma and associated with the lymphoproliferative disorders, multicentric Castleman’s disease and PEL [126,127]. No individual KSHV gene product appears to transform primary human cells by itself, but several viral proteins and non-coding RNAs have been shown to play a pivotal role in the pathogenesis of KSHV-associated tumors [6,128]. The viral proteins latency-associated nuclear antigen (LANA), vCyclin, and viral FLICE inhibitory protein (vFLIP) drive cell proliferation and prevent apoptosis, while viral interleukin 6 (vIL6), vGPCR, and ORFK1 contribute to angiogenesis and inflammation [127].

CpG methylation analysis of the human DNA in KSHV-infected cells and KSHV-associated PELs revealed both hyper- and hypomethylated promoters compared with KSHV-negative lymphoma BJAB cells. Genes encoding proteins involved in cell cycle control, signaling pathways and metastasis were differently methylated in the KSHV-positive cells and tumors compared to control cells [111,129]. Some of the genes that were hypermethylated in KSHV-infected PEL cell lines included *CDNK2A*, *CDH1* and *CDH13* (cadherin 1 and 13), *LDHB* (lactate dehydrogenase B), *HLTF* (helicase like transcription factor, a member of the chromatin remodeling SWI/SNF family), *CCND2* (cyclin D2). The authors showed that KHSV LANA recruited DNMT3A to chromatin, and induced hypermethylation and transcriptional inactivation of these genes [43,44,45]. LANA may not only repress transcription of cellular genes by inducing hypermethylation, but it may potentiate transcriptional inhibition through recruiting the transcriptional repressor methyl CpG binding protein 2 (MeCP2), which interacts with LANA [46]. Moreover, LANA could inhibit the promoter of the TGF-β type II receptor (*TGFBR2*) through inducing hypermethylation of Sp1 binding sites, thereby preventing Sp1 binding. Epigenetic silencing of this promoter contributed to the pathogenesis of KSHV-associated tumors [130]. Two other KSHV proteins interfere with DNA methylation. vIRF1 could upregulate DNMT1 expression in a STAT3-dependent manner and by inhibiting p53 [47,49]. vIL6-induced modifications in DNA methylation promoted proliferation and migration of endothelial cells [47]. Another group showed that the vIL6/STAT3/DNMT1 axis was involved in silencing expression of caveolin 1, which promoted cell proliferation, invasion and angiogenesis of endothelial cells [49]. The mechanism by which KSHV achieves hypomethylation of the host DNA is not known. Taken together, these results indicate that KSHV-triggered DNA methylation play a role in KSHV-associated cancers.

### 2.8. HBV and Host Cell DNA Methylation

It is estimated that more than 250 million people globally are chronically infected with HBV, and each year around 800,000 patients died from HBV- and HCV-related HCC. Of these, approximately 50% of are caused by HBV [131]. HBV-induced hepatocarcinogenesis occurs due to viral genome integration causing mutations and through the actions of the viral proteins, predominantly HBx (also referred to as pX), but the surface proteins preS and S also contribute to tumor development as shown by in vitro and animal studies. The mechanisms by which HBV induces HCC have been comprehensively reviewed by others [3,5,6,78,132].

Comparing the DNA methylation profile of HBV-associated HCC and HBV-negative tumors or healthy adjacent liver tissue, HBV-infected and non-infected cells, and HBx transgenic mouse model and control mice disclosed differentially methylation. Several cellular promoters were hypermethylated in the presence of HBV or HBx, including the promoters of the genes encoding cyclin-dependent protein kinases inhibitors p21^CIP1/WAF1^ (*CDKN1A*), p14^ARF^ (*CDKN2A*) and p14^INK4B^ (*CDKN2B*), cadherin 1, RASSF1A, the spleen associated tyrosine kinase SYK (*SYK*), GSTP1, the protein phosphatase 1 regulatory subunit 13B (*PP1R13B*), the tumor promotor p53 binding protein 2 (*TP53BP2*), and insulin like growth factor binding protein 3 (*IGFBP3*) [52,53,82,84,133,134,135,136,137,138]. These proteins are involved in cell cycle control, apoptosis, migration and invasion, indicating that HBV-induced silencing of these genes play a role in HCC. Some CpG islands of genes associated with HBV-induced tumorigenesis were significantly hypomethylated in transgenic mice with liver-specific HBx-expression compared to wild-type animals, illustrating that HBV infection can also upregulate gene expression by demethylating their DNA [50].

HBV seems to affect DNA methylation by several mechanisms. One study showed that HBx could cause hypomethylation through releasing DNMT3A from promoters [51]. HBx also upregulated expression of DNMT1 and DNMT3A, but repressed DNMT3B expression in liver cell lines [52]. HBx upregulated DNMT1 expression by repressing p16^INK14A^, resulting in activation of the cyclin-dependent kinase 4/6-pRb-E2F1 pathway, and ultimately in stimulation of DNMT1 expression [53]. Moreover, HBx was shown to downregulate miR-152 and miR-101, which target DNMT1 mRNA and DNMT3A mRNA, respectively, thereby increasing the levels of DNMT1 and DNMT3A [55,56]. Another study demonstrated that HBx could recruit MeCP2, which repressed transcription [52]. HBx was found to modestly suppress DNMT3A expression in mouse liver, and to cause a strong decrease in DNMT3L levels. The latter has no methyltransferease activity but stimulates the enzymatic activity of DNMT3A. The authors also showed that HBx stimulated recruitment of HDAC1 [50]. The reason for the antagonistic effect of HBx on DNMT3B expression in liver cells and in liver is not known. Other studies demonstrated that HBx did not directly influence the expression of DNMT1 and DNMT3A and of MeCP2 and MBD1, but increased their recruitment to promoters, as was shown for the *PP1R13B* and *TP53BP2* promoters [54]. Similar to the other human tumor viruses, HBV infection alters the methylation profile of the host cell DNA, resulting in up- and downregulation of cancer-related genes, which can contribute to HBV-induced hepatocarcinogenesis.

## 3. Oncoviruses and Chromatin Remodeling

### 3.1. Histone Modification and Chromatin Remodeling Machinery

Host cell DNA is packed and present in a highly organized structure called chromatin, which is a complex of DNA, histones and other proteins. Chromatin is a dynamic structure that regulates the accessibility of DNA for transcription, replication, DNA repair and recombination. Nucleosomes are the basic units of chromatin and consist of two copies of the canonical histones H2A, H2B, H3 and H4 around which DNA is twisted. The linker histone H1 is interspersed between nucleosomes. Posttranslation modifications (PTMs) of histones will affect the chromatin structure and hence the accessibility of the DNA. The most studied and best understood histone PTMs are acetylation of lysine (K) and methylation of lysine and arginine (R) residues, and phosphorylation of serine (S), threonine (T) and tyrosine (Y) [139,140,141]. Acetylation is a reversible process and is catalyzed by an histone acetylase (HAT), while an histone deacetylase (HDAC) will reverse acetylation. Acetylation of histones will neutralize the positive charges of K residues, thereby disrupting the interaction with e.g., the negative phosphate groups of the DNA. Acetylation of histones is associated with transcriptional activity, and HDAC acts as a transcriptional repressor. Multiple methylation events can occur at the same K or R residue in histones. H3K4me3 is associated with transcriptional activity, whereas high methylation levels of histone 3 at K9 and K27 and of histone 4 at K20 (H4K20me) are typical for transcriptionally repressed chromatin. Lysine methyltransferases (KMTs) and lysine demethyltransferases (KDMs) add or remove methyl groups. Phosphorylation of histones adds negative charges that undoubtfully influence chromatin structure, but the precise role of this PTM in transcription is less understood. Histone PTMs will affect nucleosome–DNA interactions, as well as histone–histone interactions and interactions with other proteins such as histone chaperones [141,142]. Histone modifying enzymes often exist in multisubunit complexes. For example, the polycomb repressive complex 2 (PRC) includes either enhancer of zeste homolog 1 (EZH1) or EZH2, and the proteins embryonic ectoderm development (EED), suppressor of zeste 12 homolog (SUZ12) and retinoblastoma-binding protein RbAp46 or RbAp48. PRC2 catalyzes H3K27me3 by the enzymatic activity of EZH1 or EZH2 [143].

Another mechanism to change the chromatin structure is by chromatin remodelers [143,144]. ATP-dependent remodelers use ATP to remodel the chromatin. Four major families of ATP-dependent remodeling complexes exist: switching defective/sucrose nonfermenting (SWI/SNF), imitation switch (ISWI), chromodomain helicase DNA-binding protein (CHD), and inositol requiring 80 (INO80). All these complexes consist of multiple proteins [145].

Perturbed histone and modifications and remodeling of chromatin are pivotal events in oncogenesis [146]. In the next section we will discuss how tumor viruses can induce histone modifications and chromatin remodeling and how this may contribute to tumorigenesis. The effects of viral oncoproteins on histone modifying enzymes and proteins of chromatin remodeling complexes are summarized in Table 2.

### 3.2. HTLV-1 and Histone Modification and Chromatin Remodeling

HTLV-1 infection can affect histone acetylation as demonstrated for the p21^CIP1^/^WAF1^ encoding gene. Expression of this cyclin-dependent kinase inhibitor was upregulated in HTLV-1 infected cells and it was shown that histone H4, but not histone H3 was acetylated [199]. Both Tax and HBZ have been shown to be involved in the regulation of histone acetylation. Tax could bind CREB-binding protein (CBP) and its paralog p300, as well as HDAC1, whereas HBZ sequestered p300/CBP [147,148,149]. Competition between HBZ and Tax for p300/CBP disrupted the interaction of Tax with p300/CBP and abrogated Tax-induced stimulation the HTLV-1 promoter [147]. As not all ATLs express Tax, but do express HBZ, HBZ may usurp p300/CBP, thereby reducing expression of cellular genes [200,201,202]. HBZ bound to and repressed activity of another HAT, lysine acetyltransferase 7 (KAT7 alias HBO1), which acetylates histones H3 and H4 [153]. Protein levels of the HDAC sirtuin 1 (SIRT1) were higher in ATL cells compared to healthy peripheral blood mononuclear cells (PBMC). Interestingly, SIRT1 inhibitors induced apoptosis of ATL cells, suggesting an anti-apoptotic action of SIRT1 [203]. The mechanism for upregulation of SIRT1 in ATL cells is not known, but SIRT1 has been shown to interact with Tax and to suppress HTLV-1 gene expression [150]. These findings suggest that interfering with HDAC and HAT may be important in the development of HTLV-1 associated ATL.

Altered histone methylation may also contribute to HTLV-1-induced cancer. The H3K27me3 pattern in ATL cells was different from normal CD4+ T cells, indicating that HTLV-1 reprograms the H3K27me3 profile. H3K27me-silenced genes included genes whose products are involved in control of cell proliferation, cell migration, transcriptional regulation, immune response and cellular metabolism [151,204]. Fujikawa and colleagues reported that the expression of all proteins that constitute the PRC2 complex were upregulated in ATL cells compared to normal CD4+ T cells, whereas downregulated genes included tumor suppressor genes, genes encoding transcription factors, histone demethylases, and other epigenetic modifiers [151]. Tax-dependent immortalized cells showed H3K27me3 reprogramming that was significantly similar to that of ATL cells, suggesting that changes in the H3K27me3 landscape are at least partially dependent on Tax. Indeed, Tax, but not HBZ, stimulated EZH2 promoter activity in a MAPK- and NFκB-dependent manner, increased EZH2 protein levels and interacted with EZH2. Moreover, the authors showed that inhibition of EZH2 prevented Tax-dependent growth and immortalization of Tax-transfected PBMC [151]. Taken together, Tax/EZH2-dependent epigenetic modifications contribute to altered gene expression and to the survival of HTLV-1-infected cells. Tax protein induced transcription of the Ellis Van Creveld 1 (*EVC1*) and *EVC2* genes though stimulating histone H3 acetylation and H3K4me3 [205]. The EVC1 and EVC2 proteins are positive modulators of the Hedgehog signaling pathway and aberrant activation of the Hedgehog signaling is an oncogenic pathway in many types of cancer [206]. Mukai and Ohshima demonstrated that HBZ interacted with centromere protein B (CENP-B), a protein that enhances H3K9me3 by recruiting the histone methyltransferase KMT1A/SUV39H1. The interaction between HBZ and CENP-B impaired recruitment of KMT1A and significantly reduced the amount of H3K3me3 [154]. Transcription of the BCL2 like 11 (*BCL2L11*) gene, which encodes the proapoptotic protein BCL2 interacting mediator of cell death (BIM), was decreased in ATL cells compared to HTLV-negative T cell lines and normal PBMC. Ectopic expression of HBZ in T cells inhibited transcription of the BCL2L11 gene. The authors showed that HBZ-mediated repression of *BCL2L11* transcription involved inactivation of the transcription factor Forkhead box O3A (FOXO3A), hypermethylation, upregulation of H3K9me2 and H3K27me3, and reduced acetylation of histone H3. HBZ-mediated silencing of BIM expression led to decreased apoptosis and may thus contribute to HTLV-1 induced oncogenesis [207].

Two studies demonstrated that HTLV-1 could induce chromatin remodeling. The integrated HTLV-1 genome bound CCCTC-binding factor (CTCF), a chromatin remodeling protein and regulator of transcription. Recruitment of CTCF by HTLV-1 provirus may spread abnormalities in the chromatin structure of host cells, thereby affecting gene expression [155]. Mass spectrophotometry and immunoprecipitation studies showed that Tax could interact with the SWI/SNF components BRM/SWI2-related gene (BRG1) and the BRG-associated factors BAF53, BAF57, and BAF155. Tax recruited BRG1, the ATPase subunit of the SWI/SNF chromatin remodeling complex, to the HTLV-1 promoter and cellular promoters and induced acetylation of histone H4, thereby stimulating the HTLV-1 promoter activity [152]. Interestingly, HBZ displaced BRG1 from the HTLV-1 promoter. Similar to p300/CBP, Tax and HBZ compete for BRG1, thereby activating or repressing promoters. The opposite roles of Tax and HBZ in viral expression may be important for maintaining viral latency and persistence, which may ultimately lead to the development of ATL [208].

### 3.3. HCV and Histone Modification and Chromatin Remodeling

HCV can modulate histone acetylation as shown for secreted frizzled related protein 1 (*SFRP1*) promoter. The core protein was shown to downregulate SFRP1 expression by an epigenetic mechanism. The core protein increased the levels of DNMT1 and HDAC1 and stimulated their binding to the *SFRP1* promoter. This resulted in hypermethylation and reduction in histone H3 acetylation. Silencing of SRFP1 led to deregulated activation of the Wnt signaling pathway and may thus contribute to HCC-induced HCC [156]. 

HCV infection is associated with changes in histone methylation. Ectopic expression of the entire HCV polypeptide resulted in a significant loss of H4K16ac, H4R3me2, and H4K20me3, and was correlated with the altered expression of genes important in hepatocarcinogenesis such as avian myelocytomatosis viral oncogene homolog (*c-MYC*), *PTEN*, *CDH1*, epidermal growth factor (*EGF*), *CDKN2A*, and *IGFBP3* [158]. Increased protein phosphatase A catalytic subunit alpha (PPP2CA) levels and reduced H4R3me2 were observed in HCV-positive HCC tumor samples compared to matching non-tumor liver tissue. The authors showed that altered H4R3me2 was caused by PPP2CA-mediated inactivation of protein arginine methyltransferase 1 (PRMT1) [158]. HCV infection of the Huh7.5 cell line resulted in significant enrichment of the transcriptional active chromatin labels H3K9ac and H3K4me, and of the transcriptional silent chromatin marker H3K9me3, but not of H3K27me3. Infection of primary human hepatocytes or the Huh7.5 cell line was associated with reprogrammed gene expression, which can be linked to HCV pathogenesis [209]. The authors also demonstrated that once epigenetic changes had occurred, this specific gene expression pattern is maintained in cells cured for HCV infection by direct acting antivirals treatment. Thus, the presence of the virus seems no longer required for its oncogenic effects on the host cells, supporting a hit-and-run mechanism. HCV can also alter the ubiquitination pattern of histones and this may affect transcription as exemplified for several homeobox (*HOX*) genes. Kasai et al. reported that the expression of several *HOX* genes was induced in HCV infected or core protein expressing cells. HCV and core protein stimulated *HOX* gene expression by impairing histone H2A monoubiquitination via degradation of PRC1 component E3 ligase RNF2 (ring finger protein 2) [157]. As HOX proteins are associated with tumorigenesis, HCV-regulated expression of these genes may contribute to HCV-induced hepatocarcinogenesis.

### 3.4. MCPyV and Histone Modification and Chromatin Remodeling

The LTs of the murine and SV40 polyomaviruses were found to bind to, and to upregulate the expression and the activity of p300/CBP [210,211,212,213]. Whether MCPyV LT possesses similar properties has not been investigated. Busam and colleagues evidenced a strong reduction of H3K27me3 staining in virus-positive MCCs compared with virus-negative tumors. This observation suggests that epigenetic deregulation may play a role in the pathogenesis of Merkel cell polyomavirus associated MCC, but the mechanism for MCPyV-induced reduction in H3K27me and the biological significance remain to be solved [214]. Cheng and coworkers showed that sT interacted with MYCL and together they recruited the EP400 HAT and chromatin remodeling complex and bound to specific cellular promoters to stimulate their activity. One of the upregulated genes was *KDMA1*, indicating that sT may affect histone methylation. sT:MYCL:EP400 complex formation was required to transform IMR90 human diploid fibroblasts, suggesting that complex formation is important in the development of MCPyV-positive MCC [159].

### 3.5. HR-HPV and Histone Modification and Chromatin Remodeling

Several studies have shown that HATs and HDACs can play a role in HR-HPV associated cancers. Expression levels of HDAC1 and HDAC1 were increased in invasive HPV-positive cervical cancers compared normal epithelium and inversely correlated with p21^CIP1/WAFf1^ levels. RNA interference-mediated silencing of HDAC2 in HPV18-positive HeLa cells increased expression of the p21^CIP1/WAFf1^ tumor suppressor and stimulated apoptosis [171]. It is not known whether HPV oncoproteins promote HDAC1/2 expression, but it could be a strategy of the virus to prevent apoptosis. E6 of HR-HPV16, but not of LR HPV6, binds and inhibits HAT activity of p300 and CBP, whereas binding of E7 to p300/CBP stimulated their activity [160,166]. E7 also interacted with lysine acetyltransferase 2B (KAT2B; also known as p300/CBP-associated factor PCAF) and reduced its ability to acetylate histones in vitro [167]. The interaction of E6 and E7 with these HATs has been demonstrated to downregulate expression of interleukin 8 (IL-8), which is a chemotactic factor for immune cells. Hence, E6/E7-mediated downregulation of IL-8 may help HPV-infected cells to evade the immune system. The HAT TIP60, which acetylates histone H4, was targeted for proteasomal degradation by E6 and reduced acetylation of histone H4 was observed in HPV-positive cell lines compared to control cells [161]. TIP60 also helps to recruit the transcriptional repressor bromodomain containing 4 (BRD4) and is involved in DNA damage response and apoptosis. Hence, E6-induced TIP60 destabilization may relieve gene expression, abrogate DNA repair, and prevent apoptotic pathways, thereby contributing to HPV-induced carcinogenesis [215].

HR-HPV E7 was shown to interact with Mi2β, HDAC1 and HDCA2, which are constituents of the NuRD complex, a CHD chromatin remodeling complex. HPV E7 could through this interaction downregulate expression of proteins involved in immune responses and promote cell growth [168,216]. Furthermore, E7 binds BRG1, a component of the chromatin remodeling SWI/SNF complex. This interaction overcomes repression of the FBJ murine osteosarcoma viral oncogene homolog (*c-FOS*) gene transcription. Hence, E7-mediated upregulation of c-FOS protein levels may contribute to deregulation of cell cycle control [169].

HR-HPV can affect histone methylation by several mechanisms. The PRC2 complex mediates H3K27me3, which is associated with transcriptional repression. Subsequently, PRC1 binds to H3K27me-marked chromatin and further silences gene expression by monoubiquitinating lysine 119 of histone H2A. PRC2 contains the histone methyltransferase EZH2, which catalyzes mono-, di-, and trimethylation of H3 [217,218]. Perturbed H3K27me is a common histone modification in many different cancers, including HPV-positive cancers [146,219]. HPV16 E6/E7 transformed primary human skin fibroblasts had increased expression of EZH2 and reduced global H3K27me3 levels compared to normal keratinocytes. Increased EZH2 levels and the loss of H3K27me3 was also observed in HP16-positive high-grade cervical intraepithelial lesions compared to matched normal tissue. E6 and E7 were shown to stimulate expression of EZH2. E6 enhanced the levels of transcription factor FOXM1, whereas E7 activated E2F1 by binding pRb. FOXM1 and E2F1 bind the EZH2 promoter and enhance transcription [162]. Furthermore, it has been shown that p53 represses expression of EZH2, suggesting that increased expression of EZH2 may be mediated through E6-mediated loss of p53 [165]. It is somewhat paradoxical that the HPV oncoproteins upregulate expression of EZH2, while a decrease in H3K27me is observed. One explanation is that KDM6A and KDM6B, which demethylate H3K27me3, were also upregulated in E6/E7 transformed primary human skin fibroblasts cells and these may counteract the effect of EZH2. Reduced H3K27me3 and increased EZH2, KDM6A, KDM6B levels were also observed in primary human foreskin keratinocytes expressing HPV16 E7 compared to control cells [170]. The PRC1 protein B lymphoma murine leukemia virus insertion region 1 (BMI1), which recognizes H3K27me3 and stabilizes this repressive methylation mark, was downregulated in E6/E7 transformed cells [165]. This may also explain the diminished H3K27me3 levels, despite increased EZH2 levels. Moreover, phosphorylation of EZH2 by AKT negatively regulates EZH2′s enzymatic activity and E6/E7 induces EZH2 phosphorylation by AKT [165], so that the levels of EZH2 may be high, but the protein is inactive. E6/E7 modulation of EZH2, BMI1, and KDM6A levels resulted in significantly reduced H3K27me3 levels of the promoters of *HOX* genes. In accordance with cervical cancer, expression of these genes was upregulated in the E6/E7 transformed fibroblasts and in E7-expressing keratinocytes cells compared to control cells [165,170]. E6 stimulates *hTERT* promoter activity by increasing H3K4me3 and H3K9ac, which are transcription activation modifications, and decreasing methylation of the transcription repressive modification H3K9me2 [163]. HPV16-positive CaSki cervical cancer cells had lower levels of KDMC5 than HPV-negative C33A cervical cancer cells. E6 was shown to interact with histone H3K4 demethylase KDM5C and promote proteasomal degradation. The authors demonstrated that CaSki cells, which overexpressed KDMC5, grew slower and invasion and migration were reduced compared to control cells. A mouse xenograft model showed that tumors derived from CaSki-KDMC5 cells grew more slowly than CaSki-derived tumors [220]. E6 could inhibit the enzymatic activity of CARM1 (as known as PRMT4), PRMT1, and the lysine methyl-transferase KMT5A. Inhibition of the methyltransferase activity of these enzymes hampered histone methylation at p53-responsive promoters and prevented the binding of p53, hence suppressing p53-mediated transcription [164].

In conclusion, changes in histone acetylation and methylation resulted in dysregulation of cellular gene expression and may contribute to HPV-induced oncogenesis.

### 3.6. EBV and Histone Modification and Chromatin Remodeling

Increased histone acetylation and increased cellular gene expressed were observed in EBV-transformed lymphoblastoid cell lines compared to control cells [175]. EBNA2 was shown to interact with and stimulate the activity of the HATs p300, CBP, and KAT2B/PCAF, suggesting a role for EBNA2 in regulating histone acetylation [173]. EBNA3C bound p300 but interacted with also HDAC1 and HDAC2 and downregulated EBNA2-induced HAT activity [175,176]. This suggests that EBNA3C may counteract the EBNA2-induced histone acetylation by sequestering p300 and recruiting HDAC. However, EBNA2 and EBNA3C are not typically expressed in EBV-positive Burkitt’s lymphoma, gastric cancer and most nasopharyngeal carcinomas, suggesting that their role in epigenetic changes in the cancer cell may be limited. Two viral proteins that can interfere with histone acetylation are BRLF1 and BZLF1, which were found to recruit CBP [177,179]. The human genome contains almost 200,000 putative BZLF1 binding sites, suggesting that appropriation of CBP by BZLF1 may repress transcription. Indeed, induced expression of BZLF1 in EBV-negative cells caused only minor, whereas overexpression of BZLF1 in latently infected B cells provoked profound reduction in gene expression and decreased open chromatin structure ([221] and references therein).

EBV infection was also associated with changes in histone methylation. EBV infection of nasopharyngeal epithelial cells reduced the transcriptional activation mark H3K4me3 and enhanced the suppressive mark H3K27me3 at the promoter regions of several genes, including 16 DNA damage repair genes. The reduced DNA repair ability in EBV-infected nasopharyngeal epithelial cells may play an important role in nasopharyngeal carcinoma [222]. Infection of B cells with EBV resulted in a loss of H3K9me3, H3K27me3, and H4K20me3, histone markers that are associated with histone condensation. Reduction of these markers was linked to increased chromatin accessibility and gene expression, including genes involved in hallmarks of cancer such as cell cycle regulation and apoptosis, and was associated with transformation. Similar decrease in H3K9me3, H3K27me3, and H4K20me3 patterns was also obtained with LMP1 and EBNA2 deficient mutant viruses, suggesting that these proteins are not required [223]. Histone modification and chromatin remodeling seems also involved in EBV-induced pathogenesis. Schaeffner and her coworkers reported that the EBV transcription factor BZLF1 interacted with the chromatin remodeling proteins SNF2h and INO80 and this led to increased chromatin accessibility on the EBV genome [178]. EBNA-LP and EBNA2 could also associate with the INO80 complex [174]. Whether the interaction of these viral proteins with chromatin remodeling complexes affects the chromatin structure of host cells was not investigated. Another study showed that EBNA2:SNF complex was recruited to the cellular Fc fragment of IgE receptor II (*FCER2* or *CD23*) promoter [224]. It was previously demonstrated that EBNA2 stimulates CD23 expression [225], suggesting the EBNA2-mediated recruitment of SNF may be involved. The SNF2 member lymphoid-specific helicase (LSH) is overexpressed in EBV-positive nasopharyngeal tumor samples compared to EBV-negative samples, but the biological relevance was not investigated [180].

Taken, together, EBV-induced histone modifications and chromatin remodeling may be a potential cancer driver in EBV-related tumors.

### 3.7. KSHV and Histone Modification and Chromatin Remodeling

KSHV-infected cells displayed changes in the level of H3K27me3 at promoters of genes encoding proteins relevant in KSHV-induced carcinogenesis such as vascular endothelial growth factor (VEGF), p53, and toll-like receptors (TLRs) [226]. Several KSHV proteins have been shown to interfere with histone modifying enzymes and proteins of chromatin remodeling complexes. Viral interferon regulatory factor (vIRF) was shown to interact with the HATs p300 and CBP and inhibited their activity. These interactions resulted in altered chromatin structure and reduced gene expression [186]. HDAC5 lacks enzymatic activity but can be phosphorylated and transported to the cytoplasm. This will ultimately lead to anti-angiogenic gene expression [227]. It was demonstrated that vIRF3 interacted with HDAC5 and prevented nuclear export, thereby contributing to virus-induced lymphoangiogenesis [187]. Another viral protein, Rta, could also recruit CBP, as well as the SWI/SNF complex through interaction with the BRG1 subunit, and the transcriptional regulatory complex TRAP/Mediator. However, the effect on cellular gene expression in KSHV-induced oncogenesis remains to be determined [188]. LANA could interact with SAP30 (Sin3-associated protein), a component of the HDAC complex and with histone methyltransferase KMT1A/SUV39H1 and heterochromatin protein 1 to induce H3K9 methylation [181,182]. LANA, vIL6, and vFLIP stimulated EZH2 expression via the NFκB pathway. KSHV induced expression of the H3K27-specific methyltransferase EZH2 of the PRC2 complex promoted production of the proangiogenic factor ephrin-B2, indicating that EZH2 is essential for KSHV-induced angiogenesis [183,186]. Moreover, LANA was found to associate with H3K4 methyltransferase KMT2F/SETD1A and to bind the members of the chromatin modulator family BRD/BET [184,185], indicating that LANA can modify chromatin structure. However, LANA chromatin-immunoprecipitation techniques showed that LANA predominantly bound to sites that were already in an open chromatin formation and most transcription of the genes located close to LANA binding sites did not change significantly. However, LANA may induce gene-specific chromatin changes as demonstrated for some interferon gamma (IFNγ)-responsive genes [128]. LANA was found to induce sumoylation of Sp100, a component of ND10 nuclear bodies, resulting in release from chromatin and this coincided with acquisition of H3K27me3 marks [228]. KDM6B is overexpressed in several EBV-positive tumors and KDM6B expression was induced in LMP1-transfected in germinal centre B cells [172]. In conclusion, several KSHV proteins may induce histone modifications and chromatin rearrangements, thereby contributing to oncogenesis.

### 3.8. HBV and Histone Modification and Chromatin Remodeling

HBx protein of HBV was shown to activate or repress cellular gene expression. This opposite effect depended on whether HBx attracted HATS or HDACs to the promoter. HBx stimulated CRE binding protein (CREB)-dependent transcription by recruiting p300/CBP. Induction of CREB target genes may play a role in the development of HCC associated with HBV infection [189]. HBx also increased histone acetylation on the DNMT1, DNMT3A and DNMT3B promoters, thereby increasing their expression (see Section 2.8). This suggests that HBx stimulated HAT binding to these promoters [52]. HBx was shown to bind p300/CBP and to stimulate transcription of the *IL-8* and proliferating cell nuclear antigen (*PCNA*) genes. IL-8 possesses mitogenic, motogenic and angiogenic properties, whereas PCNA is implicated in DNA synthesis. Increased expression of these proteins may represent key steps in neoplastic transformation by HBV [190]. On the other hand, HDAC1, HDAC2, and HDAC3 expression was increased in HBV-positive HCCs, in HBx-expressing cells, and in the liver of HBx transgenic mice compared to matching non-tumor tissue, control liver cells, and wild-type mice, respectively [191]. HBx was shown to interact with HDAC1 and HDAC2, and HBx-induced stabilization of hypoxia-inducible factor 1 alpha (HIF-1α), a key regulator in tumor growth, angiogenesis and metastasis of HCC, involved deacetylation by HDAC1 [191,229].

HBx-caused changes in histone methylation is mediated by different enzymes. HBx stimulated the expression of the histone lysine 9-specific methyltransferase SETDB1, leading to the release of transcriptionally silenced HBV genome [193]. The effect on cellular gene expression was not examined, but upregulated expression of SETDB1 was significantly associated with HCC disease progression, cancer aggressiveness, and poorer prognosis of HCC patients [230]. HBx upregulated EZH2 expression by reducing levels of miR-101, which targets EZH2 transcripts, and by inhibiting pRb, resulting in E2F1 mediated transcription of the EZH2 gene. Furthermore, HBx increased the half-life of EZH2 [56,194,195]. HBx augmented the expression of the H3K4-specific methyltransferase set and mynd domain containing (SMYD3) and this resulted in increased transcription of the *c-MYC* proto-oncogene [196]. HBx upregulated expression of the polo like kinase 1 (PLK1). This serine/threonine kinase blocks the repressive effect of PRC2 and the transcription repression complex composed of lysine demethylase 1A (KDM1A), the co-repressor CoRest, HDAC1, and HDAC1 [192]. The KDM1A/CoREST/HDAC1/2 complex enzymatically removed histone acetylations and H3K4 methylations [231]. PLK1-mediated inhibition of PRC2 and KDM1A/CoREST/HDAC1 has been shown to stimulate the Wnt signaling pathway by increasing β-catenin expression and to promote the progression of hepatocellular carcinoma [232]. HBx was found to form a complex with the p65 subunit of NFκB, EZH2, TET2, and DNMT3L and to cause activation of the epithelial cell adhesion molecule (*EpCAM*) promoter [197]. HBx was shown to promote H3K4me3 by preventing proteasomal degradation of WD repeat domain 5 protein (WDR5), which is a core subunit of the H3K3 methyltransferase complex, and by recruiting this protein to chromatin. Silencing WDR5 expression reduced tumor formation of HBx expressing cell implanted in nude mice. These results suggest that HBx mediates its oncogenic effect in a WDR5-dependent manner [198]. 

Taken together, these findings emphasize an important role of HBV-induced histone modifications in the development of HCC.

## 4. Oncoviruses and microRNA

### 4.1. MicroRNA Biogenesis and Functions

MicroRNAs are short, non-coding RNAs that are involved in the regulation of gene expression. Most miRNA genes are transcribed by RNA polymerase II and generate an immature precursor pri-miRNA, which is processed by the RNase III enzymes Drosha and Dicer to produce mature microRNA of 21–23 bases. The mature miRNA is incorporated into the RNA-inducing silencing complex (RISC), which binds to complementary or quasi complementary sequences in the 3′ untranslated region of target mRNAs and induces their degradation or prevents their translation [233]. MicroRNAs play a pivotal role in developmental and cellular processes, but also in cancer [234]. Transcription of miRNA encoding regions is regulated by additional transcription factors and repressors, but also by DNA methylation and chromatin remodeling of their promoters. The role of some microRNAs in virus-positive cancers is outlined below and summarized in Table 3.

### 4.2. HTLV-1 and microRNA

No HTLV-1-encoded microRNA has been described so far, but HTLV-1 can alter the expression levels of cellular microRNAs. HTLV-1-transformed cells and ATL-derived cell lines had reduced levels of miR-150 and miR-223. STAT1, whose mRNA is a direct target for these miRNAs, was upregulated in HTLV-1-transformed and ATL cells and was required for the proliferation of these cells. MHC-I levels were also increased in these cells and enhanced MHC-I expression helped the tumor cell to avoid immune clearance [235]. STAT1 has been found to play a role in chromatin decondensation of the MHC locus [236], which may explain concomitant increased expression of both proteins. The mechanisms by which HTLV-1 repressed miR-150 and miR-223 expression are incompletely understood, but Tax, as well as HBZ could increase the expression and activity of E2F1, which is a repressor of the miR-223 promoter [258,259,260]. The HTLV-1 HBZ protein was also shown to affect microRNA levels. HBZ upregulated miR-17, miR-21, miR23b, and miR-27b by a posttranscriptional maturation mechanism. These microRNAs target mRNA of the nucleic acid binding protein 1 (*NABP1*) gene encoding the ssDNA binding protein HSSB2. Silencing of this DNA repair factor stimulated cell proliferation and genomic instability, indicating that HTLV-1 infection may trigger proliferation and genomic instability by the HBZ/miR-17+miR-21/HSSB2 axis [237].

### 4.3. HCV and microRNA

HCV does not seem to encode viral microRNA probably because of its cytoplasmic location, which deprives the virus from nuclear proteins, such as RNA polymerase II and Drosha, required for microRNA biogenesis. However, comparative microRNAome profiling of HBV-associated HCCs and HBV-negative HCCs, and of HepG2 hepatocytes stably transfected and full-length HCV genome and control cells demonstrated that HCV elicited changes in cellular miRNA expression [238,261,262,263]. MicroRNAs including miR-30c, miR-122, miR-124, miR-138, miR-152, and miR-203 were downregulated, whereas miR-21, miR-93, 193b, miR-196a, and miR-758 were upregulated. These microRNAs were shown to regulate cell proliferation, invasion and migration, immune evasion, immortalization and cell survival. The core protein was demonstrated to be responsible for modulating the expression of these microRNA. One modus operandi of core protein-mediated microRNA repression was by inducing methylation of microRNA genes such as the miR-124 gene. The transcript of the SMYD3 protein, a protein that stimulates migration and invasion, was shown to be a direct target of miR-124. Hence, the core protein can stimulate tumor migration and invasion by DNMT1/methylation-mediated inhibition of miR-124 expression, and consequently preventing miR-124-induced silencing of SMYD3 [262]. EZH2 was shown to be also a direct target of miR-124 and a significant inverse correlation between miR-124 and EZH2 mRNA levels was measured in HCC tissues [239]. This finding suggests that HCV core protein can affect H3K27me3 through a miR-124/EZH2 pathway. Another mechanism by which the core protein affected microRNA levels was by suppressing the activity of Dicer, thereby interfering with the biogenesis of microRNAs [264]. The non-structural proteins NS3, NS4A, NS4B, and NS5A also affected the expression of cellular microRNAs that stimulate proliferation, cell survival, migration and invasion, and immune evasion [238]. The mechanisms by which these HCV proteins modify microRNA expression remains to be determined.

### 4.4. MCPyV and microRNA

MCPyV encodes a microRNA, referred to as miR-M1, which negatively regulates the expression of LT, a viral protein involved in transcription and replication of the MCPyV genome [240,265]. This viral-encoded microRNA is predicted to regulate viral replication and promote immune evasion [240,241]. Ectopic expression of miR-M1 resulted in significant differentially expressed genes compared to control cells, especially genes whose proteins are involved in the immune response, but also in cell motility [241]. One of the confirmed miR-M1 targets was the transcript for SP100, a protein involved in antiviral immunity. MiR-M1-mediated silencing of SP100 resulted in reduced secretion of C-X-C- motif chemokine ligand 8 (CXCL8) and attenuated neutrophil migration in cell culture. These in vitro data suggest a role for miR-M1 in aiding MCPyV-positive MCCs to escape the immune system. However, deep sequencing analysis showed that very low miR-M1 levels are detectable in less than 50% of MCPyV-positive MCC tumors and undetectable in the majority of MCC tumors, jeopardizing miR-M1′s biological significance in tumorigenesis [265,266]. Minimizing the levels of miR-M1 allows the infected cell to produce more LT transcripts that can be translated into the LT oncoprotein. 

Comparative microRNAome studies between virus-positive and virus-negative MCC cell lines and tumors have identified several cellular microRNAs whose expression is associated with the MCPyV status (for a recent review see [267]). These included miR-203, miR-30a-3p, miR-769-5p, miR-34a, miR-30a-5p and miR-375 [267,268]. MiR-375 has been most extensively studies and its serum level correlates with tumor burden, demonstrating that miR-375 serum levels can be considered a valid surrogate biomarker of tumor burden in MCC patients [243,269]. However, the function of miR-375 in MCC is controversial. Abraham and colleagues described the involvement of miR-375 in neuroendocrine differentiation and knockdown of miR-375 in virus-positive cell lines did not alter their growth properties [270]. Recently, Kumar and colleagues found that MCPyV T-antigens and the MCPyV-regulated miRNAs miR-375, miR-30a-3p and miR-30a-5p suppressed autophagy by targeting multiple autophagy genes, thereby protecting MCC cells from autophagy-associated cell death [242]. LDHB is a target of miR-375. This enzyme catalyzes the conversion of lactate to pyruvate and NAD+ to NADH and is known to play important roles in cancer cell growth and progression [271,272]. In another paper, Kumar and colleagues reported that LDHB expression was inversely correlated with miR-375 levels in MCC cells and LDHB was found to have distinct roles in MCPyV positive and MCPyV negative MCC cells. In virus-associated MCC cells, inhibition of miR-375 expression reduced cell growth and induced apoptosis, and silencing of LDHB restored cell growth caused by miR-375 inhibition. An opposite effect was observed in MCPyV negative cell lines were silencing of LDHB reduced cell growth [244]. MiR-375 expression seems to be activated by transcription factor ATOH1 [96]. However, ATOH1 is downregulated during MCC progression, whereas another study demonstrated that expression of ATOH1 was increased in advanced MCCs MCPyV associated carcinogenesis [273,274]. Interestingly, ATOH1 expression is induced by ectopic expression of truncated forms of LT (which are expressed in MCPyV-positive MCCs) in fibroblasts [96]. Another study questioned the role of miR-375 in MCPyV-associated MCC. Highly effective miR-375 knockdown in virus-positive MCC cell lines did not significantly modify the cell viability, morphology and oncogenic signaling pathways [275]. Enrichment of miR-375 in extracellular vescicles has been described, suggesting a role of this microRNA in intercellular communication of MCC. Becker and his group showed that extracellular vesicle-mediated transmission of miR-375 to fibroblasts caused phenotypic changes toward cancer-associated fibroblasts. This observation suggests that miR-375 may contribute to generating a tumor microenvironment [276].

A subset of miRNAs associated with tumor metastasis and MCC-specific survival has been identified. Functionally, overexpression of miR-203 was able to inhibit cell growth, to induce cell cycle arrest, and to regulate survivin expression in MCPyV negative-MCC cells, but not in MCPyV-positive MCC cells. These findings reveal a mechanism for survivin expression regulation in MCC cells and offer insights into the role of miRNAs in MCC tumorigenesis [268].

MCPyV has also been detected in other cancer types, including non-small cell lung cancer [277]. Lasithiotaki et al. demonstrated overexpression of miR-21 and miR-376c in MCPyV-positive compared MCPyV-negative non-small cell lung cancers, whereas miR-145 levels were higher in the virus-negative tumor samples [278].

In conclusion, the MCPyV-encoded microRNA miR-M1 does not seem to be involved in MCC, but MCPyV infection modifies cellular microRNA expression, which may play a role in tumorigenesis and the tumor microenvironment.

### 4.5. HR-HPV and microRNA

MicroRNA prediction algorithms have been used to forecast putative HPV16- and HPV18-encoded miRNAs [279,280]. By using Northern blotting, a weak hybridization signal corresponding to mature HPV18-miR-LCR3 was detected in the HPV16-positive CaSki cell line [279]. This putative HPV miRNA has high sequence identity to cellular miR-466. Possible targets are genes encoding proteins involved in proliferation, transcription, signaling pathways. Whether HPV18-miR-LCR3 is a truly HPV-encoded miRNA remains to be established. The group of Auvinen identified and validated the expression of 5 HPV16-encoded microRNAs (HPV16-miR-H1, H2, H3, H5 and H6) in HPV-positive cell lines and cervical cancers. In all cases, HPV16-miRs were expressed at low levels [280,281]. Among the putative targets were mRNAs encoding proteins involved in focal adhesion, cell migration, cell proliferation and tumor suppressors [280].

Several studies have shown that HR-HPV positive tumors and cell lines expressing the HR-HPV oncoproteins E5, E6 or E7 have altered cellular microRNAomics compared to control tissue and cells. Upregulation and downregulation of cellular microRNAs have been observed. The microRNAs dysregulated in HPV-positive cervical cancers are involved in cell proliferation, cell survival, angiogenesis, invasion, and migration underscoring their role in HR-HPV pathogenesis (Table 3; [108,109,114,245,282]).

One mechanism by which HPV affected microRNAs expression was by modifying the promoter methylation pattern of the genes encoding microRNAs [246]. For example, no methylation of miR-124 promoter was found in normal cervical cancer, whereas hypermethylation level of the miR-124 promoter increased with the cancer grade [246]. Methylation of the miR-124 promoter was increased and levels of this microRNA were decreased in human foreskin keratinocytes immortalized with either HPV16 or HPV18. Concordantly, levels of insulin like growth factor binding protein 7 (IGFBP7), whose mRNA is a target for miR-124, were increased. Furthermore, ectopic expression of miR-124 in HPV16-positive SiHa and CaSki cervical cancer cell lines reduced their proliferation rate and migration capacity. These results support a role for silencing miR-124 in HPV-mediated cervical carcinogenesis. HPV-induced hypermethylation of miRNA promoters is mediated by increased DNMT1 expression and activity by E6 and E7 as discussed in Section 2.4. HR-HPV infection was also associated with reduced methylation of microRNA genes, but the mechanism by which HR-HPV decreases microRNA promoter methylation is not known [108,109,114,245,282]. Another mechanism by which HR-HPV affected microRNA expression is through targeting cellular proteins involved in the transcription of microRNA genes. HR-HPV E6 induced degradation of p53 and E7 appropriated pRb, which altered the transcription levels of microRNA-encoding genes [114]. A third mode of disturbing microRNA levels is by interfering with the biogenesis of microRNAs. HR-HPV E6 and E7 could altered the expression of microRNA processing proteins, including Drosha and Dicer and different expression of these proteins was observed in HPV-induced cancers compared to normal tissue. Dysregulation of microRNA processing proteins perturbed miRNA biogenesis and affected translation of their target mRNAs [108,283,284].

### 4.6. EBV and microRNA

The EBV BHRF1 cluster and the BamHI-A rightward transcript (BART) clusters 1 and 2 encode >40 mature miRs, which can regulate host and viral gene expression. These viral miRs are crucial for EBV-associated tumorigenesis by e.g., inhibiting apoptosis, immune evasion, and cell growth [45,247,248,249]. For example, EBV miR-BART2-5p silences MHC class I polypeptide-related sequence B (MCIB) expression to inhibit natural killer cell recognition and activation, allowing immune evasion of the EBV-positive tumor cell. Other EBV miRNAs that have a predicted role in immune evasion, include miR-BHRF1-3 (target is CXCL11, a T cell attracting chemokine), miR-BART15 (target is the inducer of pro-inflammatory cytokines NLR family pyrin domain containing 3; NLRP3 or cryopin), and miR-BART5-5p (represses the expression of the pro-apoptotic protein p53 upregulated modulator of apoptosis; PUMA). EBV miR-BART9, miR-BART 11 and miR-BART 12 inhibit apoptosis by repressing expression of BIM [247,248,249].

EBV infection also altered the expression of host cell miRNAs. Comparison of the microRNAomes from EBV-positive nasopharyngeal tissue and non-tumor tissue disclosed several cellular miRNAs that were upregulated, but also many were downregulated. One of the cellular miRNAs induced by EBV is miR-155, an oncomir crucial for B cell transformation and proliferation [248]. The microRNA profile of EBV-positive gastric cancers and EBV-positive lymphomas also displayed differentially expressed host cell microRNAs compared with virus-negative tissue. Again, these microRNAs target transcripts of proteins involved in apoptosis, immune evasion, cell proliferation, invasion and metastasis, hinting to a crucial role in the carcinogenesis of these EBV associated tumors [248,249,285,286]. EBV induced chromatin changes can also be mediated by microRNA. The EBV protein EBNA2 was found to induce miR-146-5p, which targets KDM2 mRNA [222].

The mechanisms by which EBV modulate microRNA expression have been less studied but may include changes in DNA methylation and chromatin of the microRNA genes induced by viral proteins as discussed in Section 2.6 and Section 3.6. EBV can also affect the biogenesis of microRNAs as shown for EBV miR-BART6-5p, which targets Dicer mRNA [248].

### 4.7. KSHV and microRNA

KSHV produces 25 mature microRNAs and more than 2000 host transcripts that encode proteins associated with KSHV pathogenesis can be directly targeted by these viral microRNAs [250,287,288]. The functions of KSHV microRNAs have been extensively studied and showed that they perturbed expression of host proteins, which are involved in angiogenesis, proliferation, cell survival, migration and invasion, and immune evasion [45,247,248,250,251,288]. A few examples are mentioned. KSHV miR-K12-1 helped evading cell cycle arrest by silencing p21^CIP1/WAF1^ expression. KSHV miR-K12-5, miR-K12-9 and miR-K10a/b targeted the pro-apoptotic protein Bcl-2-associated factor 1 (BCLAF1), whereas miR-K12-1, miR-K12-3, and miR-K12-4-3p suppressed caspase 3 expression. These microRNAs allowed the virus to avoid apoptosis. KSHV evaded the innate immune system by miR-K12-5- and miR-K12-9-mediated reduction of myeloid differentiation primary response 88 (MYD88) and interleukin-1 receptor-associated kinase 1 (IRAK1), respectively. Finally, KSHV microRNAs promoted angiogenesis by downregulating the levels of the anti-angiogenic factor thrombospondin, SH3 domain binding glutamate-rich protein (SH3BGR) and CD82 [247,248,252,288,289]. KSHV-encoded microRNAs were demonstrated to play a role in DNA methylation because infection with a mutant virus unable to express KSHV microRNAs resulted in almost complete loss of DNA methylation. Possible mechanisms could be through miR-K12-4-5p, a KSHV microRNA that prevented synthesis of the DNMT repressor Rbl2, and via miR-K12-11, which targets the PRC2 component Jarid2 [253]. Jarid 2 was also shown to function as a tumor suppressor and regulator of B-cell survival. Hence, KSHV miR-K12-11-mediated inhibition of Jarid2 may contribute to KSHV-induced malignant transformation [290]. 

The role of KSHV-provoked dysregulated expression of host cell microRNAs in cancer has been extensively reviewed [248,251,252]. We will briefly mention some examples. The viral protein K15 was shown to induce expression of cellular miR-21 and miR-31, which promoted cell migration, angiogenesis, and lymphangiogenesis. The viral proteins LANA and Kaposin B repressed expression of cellular miR-221 and miR-222, which resulted in increased cell migration. vFLIP upregulated miR-146a levels in an NFκB-dependent manner. This host cell microRNA silenced C-X-C motif chemokine receptor 4 (CXCR4), which promoted the premature release of KSHV-infected endothelial cell progenitors into the blood stream [248,252]. Similar to KSHV-encoded microRNAs, KSHV- induced host cell microRNAs could exert an effect on chromatin structure. KSHV was found to upregulate cellular miR-132, which targeted the HAT p300 mRNA [254]. These findings underscore a role for viral and cellular microRNA in KSHV-associated cancer.

### 4.8. HBV and microRNA

HBV encodes two viral miRNAs: HBV-miR-2 and HBV-miR-3. HBV-miR-2 may act as an oncomiR because it was found to promote cell growth, migration and invasion by downregulating the expression of the E3 ubiquitin-protein ligase tripartite motif containing 35 (TRIM35) and upregulating protein levels the GTPase RAN. TRIM35 is a proapototic protein and can inhibit the Warburg effect, whereas RAN is involved in nucleocytoplasmic transport, but also in metastasis. HBV-miR-3 enhances cell invasion and proliferation by e.g., silencing PP1A and PTEN [255,256]. 

Several studies showed a role for HBx in up- and downregulating the expression of cellular microRNAs, including miR-10, miR-132, miR-143, and miR-193b. This has been the topic of excellent reviews [190,255,257,263,291,292]. HBx modulates microRNA expression by inducing epigenetic changes in microRNA-encoding genes or modulating expression of genes whose products are involved in microRNA biogenesis. HBx can affect DNA methylation, histone acetylation and histone methylation as discussed in Section 2.7 and Section 2.8, which will affect transcription of the microRNA-encoding region. HBx can also affect the affinity of transcription factors involved in transcription of microRNA genes. For example, HBx can interfere with p53 sequence-specific DNA binding of and inhibit p53′s transcriptional activity [132], stabilize c-MYC [293], and activate NFκB-mediated transcription [294]. These three transcription factors have been shown to affect transcription of microRNA genes [295,296,297]. Moreover, HBx can repress Drosha expression leading to dysregulation of microRNA biogenesis [298]. MicroRNAs modulated by HBx were demonstrated to target genes that encode proteins involved in cell cycle progression, cell survival, immune evasion, invasiveness and migration, and angiogenesis [292]. Thus, dysregulation of microRNA expression is a pivotal mechanism by which HBV promotes hepatocellular carcinogenesis.

## 5. Oncoviruses and Long Non-Coding RNAs

### 5.1. Long Non-Coding RNA Biogenesis and Functions

Long non-coding RNAs (lncRNAs) are a heterogeneous group of RNAs that are more than 200 nucleotides long and are not translated into functional proteins. Most lncRNAs are generated by RNA polymerase II and can contain a 5′ cap and 3′ polyA tail. So far, ~18,000 lncRNA genes have been identified in the human genome, but their number is still increasing. The lncRNA genes produce almost 50,000 transcripts, but many remain to be annotated [299]. LncRNAs can act as guides for proteins, including chromatin-modifying complexes and transcriptional activators or repressors. They can also sequester microRNAs and can by binding mRNA, regulate splicing and stability, editing and subcellular localization. LncRNAs can also associate with DNA and regulate histone modification and DNA methylation. Moreover, lncRNAs can induce structural changes in proteins. Therefore, lncRNAs play crucial roles in gene expression, but they are also important in maintaining chromosome integrity. LncRNAs are crucial for normal cellular processes, but there is clear evidence that they are involved in cancer [300,301]. Some examples of lcnRNA and their role in virus-positive cancers are discussed below and are summarized in Table 4.

### 5.2. HTLV-1 and lncRNA

HTLV-1 produces the antisense mRNA HBZ that is inefficiently polyadenylated and as a result the minor fraction of properly polyadenylated HBZ mRNA is transported to the cytoplasm and translated into the HBZ protein, while the majority of aberrant polyadenylated antisense mRNA is retained in the nucleus and acts as lncRNA. Nuclear HBZ mRNA could bind to the promoters of the cellular genes, including the genes encoding C-C chemokine receptor type 4 (CCR4) and E2F1, and enhanced transcription of these genes, resulting in stimulation of proliferation of HTLV-1-infected cells. HTLV-1 antisense mRNA also promoted expression of survivin [302]. The exact mechanism by which HBZ mRNA exerts its transcriptional regulatory functions are unknown but altered gene expression by this lcnRNA can contribute to HTLV-1-induced oncogenesis.

Comparing the levels of cellular lncRNA in ATL cells, HTLV-1-infected cell lines and control cells revealed upregulation of lncRNAs ANRIL (antisense noncoding RNA in the INK4 locus), H19, and SAF (Fas-Antisense) and slight downregulation of HOTAIR (HOX antisense intergenic RNA) and TUSC7 (tumor suppressor candidate 7) by HTLV-1 [303]. The authors showed that enhancement of ANRIL expression depended on transcription factor E2F1. The exact mechanism by which HTLV-1 regulates ANRIL expression is not known, but Tax has been shown to increase expression and activation of E2F1, whereas HBZ abrogated the interaction between pRb and HDAC3, thereby activating E2F1 [260,327]. Knockdown of ANRIL in ATL cells impaired proliferation and provoked apoptosis. Tumor growth of xenografted ANRIL knockout cells was reduced compared to wild-type cells in mice. ANRIL could form a complex with EZH2 and p65 and enhanced the binding of p65 to NFκB-responsive promoters, whereas ANRIL also formed a complex with EZH2 and repressed p21^CIP1/WAF1^ expression by increasing H3K27me of the *CDKN1A* promoter [303]. In conclusion, HTLV-1-encoded lncRNA and HTLV-1-induced cellular lncRNAs are involved in processes controlling cell proliferation and cell survival and may contribute to HTLV-1 associated leukemogenesis.

### 5.3. HCV and lncRNA

So far, no HCV-encoded lncRNAs have been identified. However, it was shown that the 5′ untranslated region could be processed by the cellular endoribonuclease XRN1, generating subgenomic viral RNAs that are not translated and therefore may act as viral lncRNAs [328]. The functions of these subgenomic viral RNAs remain to be determined.

Results from several studies comparing HCV-positive HCC with healthy liver tissue showed that several lncRNAs have significantly different expression levels [291,304,305,329]. Several of these HCV-induced lncRNA affect the viral life cycle and are beyond the scope of this review [305,329]. However, other HCV-induced lncRNAs are related to HCC, and while the function of most of these lncRNAs remains elusive, the role of some lncRNAs in HCV-related HCC has been addressed. LncRNA urothelial carcinoma associated 1 (UCA1) is involved in epithelial-to-mesenchymal transmission through sponging miR-203, resulting in increased SNAI2 expression levels. PVT1 (plasmacytoma variant translocation 1) lncRNA recruits EZH2, which facilitates histone modification. PVT1 could promote HCC cell proliferation by stabilizing nucleolar protein 2 and by downregulating transcription of the proto-oncogene c-myc. AK021443, LINC01419 (or PRLH1 for p53-regulated lncRNA for homologous recombination repair 1), and HULC (highly upregulated in liver cancer) lncRNAs are upregulated and stimulate cell proliferation or metastasis. AF070632 and aHIF (antisense to hypoxia-inducible factor 1 alpha) lncRNAs are downregulated. The former is involved in cell metabolism, while the function of the latter is unknown [304,305].

### 5.4. MCPyV and lncRNA

The existence of putative MCPyV-encoding lncRNAs and the effect of MCPyV infection on host cell lncRNA expression have not been addressed. One study in head and neck squamous cell carcinoma cells demonstrated that miR-375 silenced the expression of the glucose transporter protein solute carrier family 2 member 1 (SCL2A1 or glucose transporter type 1; GLUT1) by targeting SCL2A1 mRNA. The head and neck squamous cell carcinoma glycolysis-associated 1 (HNGA1) lnc RNA was upregulated and HNGA1 functioned as a sponge for miR-375. Ectopic expression of lcnRNA HNGA1 in head and neck squamous cell carcinoma cells stimulated cell proliferation and glycolysis, and accelerated tumor growth in xenograft mouse [306]. The expression level of lcnRNA HNGA1 in MCC has to the best of our knowledge not been examined, but miR-375 is upregulated in MCC (see Section 4.4), suggesting that little or no HNGA1 lcnRNA is present in MCC tumors. MCPyV sT was shown to upregulate the expression of the glucose transporters SLC2A1 and SLC2A3 in normal human fibroblasts and SCL2A1 is significantly expressed in MCC [330,331,332]. These findings suggest that MCPyV uses an HNGA1-independent mechanism to upregulate expression of SLC2A1.

### 5.5. HPV and lncRNA

Several lncRNAs previously found to be involved in cancer are also differentially expressed in cervical cancers compared to control samples. RNA interference-mediated silencing of E7 in HPV18-positive HeLa cells or expression of E6 in primary human keratinocytes resulted in altered expression of several lncRNAs compared to control cells [114,245,309,333,334]. One of the upregulated lncRNA in cervical cancers is HOTAIR. This lncRNA could sponge miR-23b and miR-143-3p, could recruit EZH2 and promoted expression of VEGF and matrix metalloproteinase 9 (MMP9), thus stimulating carcinogenic processes [245]. Another lncRNA that was upregulated in cervical cancers is thymopoietin pseudogene 2 (TMPOP2) lncRNA. p53 bound the promoter region of the *TMPOP2* gene and inhibited its expression. E6 released p53-mediated inhibition of TMPOP2 expression. Interestingly, overexpression of TMPOP2 enhanced E6 and E7 protein levels because TMPOP2 sponged miR-375 and miR-139, which target E6/E7 mRNA [307]. Moreover, TMPOP2 inhibited E-cadherin expression by recruiting EZH2. Thus, enhanced expression of TMPOP2 seems to play an important role in HPV-induced tumorigenesis. E6 and E7 downregulated expression of cervical cancer DHX9 suppressive transcript (CCDST) lncRNA, which resulted in increased DExH-box helicase 9 (DHX9) protein levels, thereby accelerating cell mobility and angiogenesis [308]. E7 and to a lesser extent E6 elevated Fanconi anemia complementation group 1–2 (FANCI-2) lncRNA levels and this was dependent on YY1 binding sites in the promoter region of *FANC-2*. E6 and E7 were found to reduce miR-29a levels, which targets transcription factor YY1 and E7 altered the activity of YY1 [309]. The exact role of FANCI-2 in HPV-induced cancer remains elusive. Altered lncRNA expression was also observed in HPV-positive head and neck squamous carcinomas compared with normal tissue [333]. In conclusion, affecting cellular lncRNA expression may be a mechanism that contributes to HPV-induced carcinogenesis.

### 5.6. EBV and lncRNA

EBV encodes two small non-coding RNAs EBV-encoded RNA1 (EBER1) and EBER2 of 167 and 172 nucleotides long, respectively. Both are RNA polymerase III transcripts and they are present in high copy numbers (~10^6^ for EBER1 and ~2.5 × 10^5^ for EBER2) in infected cells. Although shorter than classical lncRNAs, EBERs are considered lncRNAs. EBER2 acts as a guide RNA to recruit transcription PAX5 to viral target sites, where the complex suppresses transcription. The role of EBER1 remains poorly understood, but both EBER1 and EBER2 play a role in suppressing the innate immune system, avoiding apoptosis and activating the oncogenic phosphatidylinositol 3-kinase (PI3K)-Akt signaling pathway [45,310,311]. A recent study reported that extracellular vesicles could transmit EBERs from EBV-positive nasopharyngeal carcinoma cells to endothelial cells and promote angiogenesis through upregulation of vascular cell adhesion molecule 1 (VCAM1) expression via TLR3/retinoic acid-inducible gene 1 protein (RIG-1)/MAPK pathway [335]. Taken together, EBER1 and EBER2 play a crucial role in EBV-induced tumorigenesis. EBV encodes other lncRNAs, including BART transcripts, and the EBV BamHI leftward reading frame 1 (BHLF1) region. The latter encodes also and circular RNA (see Section 6.6). BART lncRNAs are involved in the epigenetic regulation of host gene expression and were demonstrated to inhibit expression of interferon beta 1 (*IFNB1*) and *CXCL8* genes. BHLF1 lncRNAs promote EBV replication but may also contribute to viral latency [312,313,314].

Comparison of EBV-positive tumors with control cells identified several cellular lncRNAs that were differentially expressed. Some of these cellular lncRNAs, such as H19, HOTAIR, and metastasis-associated lung adenocarcinoma transcript 1 (MALAT1, aka nuclear-enriched abundant transcript 2; NEAT2) interfere with processes such as apoptosis, migration and invasion, proliferation, and DNA repair [45,313,314,315]. In conclusion, both EBV lncRNAs and EBV-induced cellular lncRNAs play indispensable roles in EBV-provoked malignancies.

### 5.7. KSHV and lncRNA 

Approximately 16 potential KSHV lncRNAs have been described, with polyadenylated nuclear RNA (PAN) lncRNA the most important and best-characterized. This lncRNA is involved in viral gene expression, replication, and immune modulation. PAN RNA was shown to bind transcription factor interferon regulatory factor 4 (IRF4) and inhibit transcription of IRF4-responsive genes. Moreover, PAN lncRNA could interact with H3K27-specific demethylases UTX and JMJD3, and with the PRC2 components EZH2 and SUZ12. Hence, PAN lncRNA affects chromatin modification, resulting in altered host gene expression and seems to be required for efficient nuclear export of mRNA [45,128,314,316]. This viral-encoded lncRNA may play an essential role in KSHV-induced cancers.

Several cellular lncRNAs, including H19, growth arrest specific 5 (GAS5), deleted in lymphocytic leukemia 2 (DLEU2) and MALAT1 are abnormally expressed in KSHV-infected cells and aberrant expression of these lncRNAs has been associated with oncogenic processes such as proliferation, migration, invasion, cell survival and angiogenesis [314]. KSHV can also provoke changes in DNA methylation and histone modifications through induction of cellular lncRNA as shown by Yang and coworkers. The authors identified KSHV-induced KDM4A-associated transcript (KIKAT/LINC01061) lncRNA as an KSHV-induced cellular lncRNA and demonstrated that this lncRNA could interact with the histone lysine demethylase KDM4A, thereby providing an open chromatin environment and allowing activation of gene transcription. Expression of 44 genes was upregulated and some of these have been identified in cancer-related pathways. One of them was *ATOM*, which encodes angiomotin, a protein that promotes cell migration and angiogenesis. In accordance, overexpression of KIKAT/LINC01061 in SLK cells induced cell migration [317]. Hence, KSHV-induced expression of KIKAT/LINC01061 may play a role in KSHV’s pathogenicity.

### 5.8. HBV and lncRNA

The expression of several lncRNAs are dysregulated in HBV-associated HCC compared to healthy liver tissue. Some of these lncRNAs are discussed here. For a complete overview, the reader is referred to excellent recent reviews [291,305,318,319,321,322,326].

Examples of cellular lncRNAs upregulated by HBV include highly expressed in HCC (HEIH), UCA1, HOTAIR, HULC, and LINC00152, which all were shown to interact with EZH2 and repress gene expression, thereby promoting proliferation, migration and invasion, cell survival, and tumor growth. UCA1 recruited EZH2 to the *CDKN1B* promoter, whose gene encodes cyclin dependent kinase inhibitor p27^KIP1^, and repressed transcription. Another lncRNA that is upregulated is ANRIL, which binds PRC2 and represses transcription of Krüppel-like factor 2 and sequesters miR-122-5p. Knockdown of ANRIL expression induced apoptosis and suppressed proliferation, invasion and migration of HCC cells in vitro. Integration of HBV adjacent to long interspersed nuclear element 1 (LINE1) resulted in the transcription of a chimeric lncRNA, HBx-LINE1, which can be detected in ~23% of HBV-associated HCC tumors. HBx-LINE1 activated the Wnt signaling pathway by inducing nuclear localization of β-catenin, and stimulated cell proliferation and metastasis in vitro. An HBx-LINE1 transgenic mouse model revealed that the animals were more susceptible to diethylnitrosamine-induced tumor formation than wild-type mice and nuclear localization of β-catenin in hepatocytes of the transgenic animals was observed [305,319,321,322,323,326]. 

Reduced expression of the lncRNAs n346077 and downregulated expression by HBx (DREH) was observed in HBV-positive HCC included. DREH inhibited proliferation and n346077 prevented migration and invasion in vitro [305,319,321,322,323,326].

The mechanisms by which HBV modulates expression of lncRNAs remains largely unknown. Studies with HBx expression in liver cell lines and with an HBx transgenic mouse model showed that HBx is directly involved in regulating lncRNA expression. HBx increased expression of HULC and HEIH [319,320,324]. HBx-induced upregulation of HULC is mediated by CREB, whereas HBx-induced expression of HEIH is mediated by transcription factor Sp1 [319,324]. HBx has been shown to increase the DNA binding affinity of CREB and to induce phosphorylation of Sp1 and augment binding to DNA [336,337], suggesting that HBx promotes transcription of the HEIH gene through CREB and Sp1. The gene of lncRNA LINC00152 was shown to be hypomethylated and expression levels correlated with HBx expression levels in tumors and was induced when HBx was ectopically expressed or downregulated when HBx was silenced in vitro [325]. As mentioned in Section 2.8, HBx can cause hypomethylation through releasing DNMT3A from promoters [51]. HBx has also been found to interact with lncRNA DLEU2 and to displace EZH2 from both the viral and host genome [338]. Taken, together, HBV-induced changes in lncRNA expression may assist with the development of HCC.

## 6. Oncoviruses and circRNAs

### 6.1. Circular RNA Biogenesis and Functions

Circular RNAs (circRNAs) are single-stranded, RNAs produced from pre-mRNA by a back splicing mechanism in which the 5′ and 3′ termini are covalently joined. They vary in length from less than a hundred to several thousands of nucleotides [339,340]. CircRNAs function in transcriptional, post-transcriptional, translational and post-translational processes by acting as miRNA/protein sponges, modulators of splicing, and by recruiting proteins and affecting protein function and stability. They can also serve as mRNA that are translated into peptides [340,341]. Compiling evidence that underscores their role in cancer was the topic of recent reviews [342,343,344]. Some examples of circRNAs that are encoded and induced by tumor viruses are discussed below and are summarized in Table 5.

### 6.2. HTLV-1 and circRNA

The existence of HTLV-1 encoded circRNA and whether HTLV-1 can induce the production of cellular circRNA have not been investigated. However, after converting its ssRNA virus genome into dsDNA, the dsDNA integrates into the host chromosome and RNA polymerase II-derived viral transcripts are spliced, suggesting that viral circRNA may be produced.

### 6.3. HCV and circRNA

It is still unclear whether HCV encodes circRNA. The fact that this virus replicates in the cytoplasm, in the absence of the nuclear splicing machinery, may explain why no HCV circRNA is generated. A recent study examined the cellular circRNA profile in uninfected and HCV-infected liver cells. The authors found 10 circRNAs that were significantly upregulated, whereas 63 had decreased levels in the HCV-positive cells compared to control cells. The authors elaborated on the role of the upregulated circPSD3, which was generated by a backsplicing event between exon 5 and exon 8 from the pleckstrin and Sec 7 domain containing (PSD) transcript and found that depletion of circPSD3 diminished viral infectivity [345]. The mechanism by which HCV dysregulates expression of cellular circRNA and a possible role of circPSD3 and the other circRNAs in HCV-induced HCC remain to be explored.

### 6.4. MCPyV and circRNA

The LT and sT encoding region of MCPyV contains an alternative reading frame which encodes the ALTO protein with unknown function [359]. A recent study identified two viral circRNAs derived from ALTO mRNA, circALTO1 (762 nucleotides in length) and circALTO2 (940 nucleotides long) in MCPyV-positive MCC cell lines, whereas only circALTO2 was detected in virus-positive tumors, suggesting that the abundance of the circALTO isoforms might differ in vivo [346]. The circALTOs were stable, predominantly located in the cytoplasm, and enriched in exosomes. CircALTOs were also N6-methyladenosine (m6A) modified, which has been reported to promote cap-independent translation [360]. Indeed, circALTO but could be translated and was negatively regulated by MCPyV miR-M1. Transfection of cells with expression plasmids for circALTO1 or circALTO2 showed that ALTO stimulated the SV40 early and late promoter, and the cytomegalovirus immediate early promoter, but not the MCPyV early and late promoter, the Trichodysplasia spinulosa polyomavirus promoter, nor two cellular promoters (the elongation factor 1-alpha and the phosphoglycerate kinase 1 promoter). These findings suggest that ALTO functions as a transcriptional activator for some promoters. Accordingly, overexpression of circALTO1 resulted in significant upregulation of a large number of genes, while only a few genes were markedly downregulated. Proteins encoded by these genes included components of NFκB signaling pathway, transcription factors, and inflammatory and anti-viral cytokines, suggesting the circALTO can modulate genes and pathways implicated in MCPyV pathogenesis. As circALTOs are enriched in exosomes, it is tempting to the speculate that circALTOs could prepare recipient cells for MCPyV infection and promote tumor development [346]. MCPyV may encode additional circRNAs because potential circRNAs were predicted upstream of genes encoding the capsid protein VP2 [346]. Another study identified a 762 nucleotide long circRNA, which the authors designated circMCV-T, in MCPyV-positive MCC cell lines and tumor samples [347]. This circMCV-T was unlikely to be translated but may act as a decoy for the MCPyV-encoded microRNA miR-M1. MiR-M1 targets LT transcripts and the authors showed that circMCV-T sequestered miR-M1, thereby reversing the inhibitory effect of miR-M1 on LT expression. The authors predicted that circMCV-T may aid viral replication by sequestering miR-M1 from the viral transcripts encoding the proteins involved in replication. Indeed, they showed that exogenous expression of circMCV-T was accompanied by increase in the levels of LT and sT transcripts and stimulated viral replication. The complex interaction between viral mRNA, miRNA and circRNA is important to meticulously fine-tune viral replication. A possible role for cricMCV-T in MCPyV-induced MCC, where miR-M1 levels are undetectable or very low [265,266], remains to be addressed.

### 6.5. HR-HPV and circRNA

A number of HPV-encoded circRNAs have been identified in HPV positive cervical cancers. Among these viral circRNAs (v-circRNA), is circE7 the most abundant and can be translated into E7. Multiple microRNA binding sites have been identified on circE7, suggesting that it can act as a miRNA sponge. circE7 has been reported also in HPV-positive anal and head and neck cancers [348,349]. Surprisingly, circE7 levels correlated with improved survival of patients with HPV-positive cervical and anal squamous cell carcinoma [349]. The role of other HPV-encoded circRNAs is unclear, but it is assumed that by sequestering microRNA they promote tumorigenic processes such as proliferation, cell survival, invasion, migration, and angiogenesis [245,333,348,361,362].

High throughput RNA sequencing studies of HPV-positive anogenital and oropharyngeal cancers and matched adjacent non-tumor tissues discovered numerous differentially expressed cellular circRNA [114,363,364,365]. Ectopic expression of HPV16 E7 in the HPV negative cervical cancer cell line C33A resulted in upregulation and downregulation of numerous host cell circRNAs. Upregulated circRNAs included circRNA8924, which target miR-518-d-5p/miR-519-59, and hsa_circ_0005576, which usurps miR-153-3p [350]. These circRNAs have been shown to promote proliferation, migration or invasion [350]. Hsa_circ_0018239 is also overexpressed in cervical cancer and knockdown of this circRNA suppressed migration, proliferation and immune evasion. Other studies demonstrated enhanced levels of circRNAs that target *TP53* (circ_0000263) or *SNAI2* (circ_000284) mRNA. The latter encodes a protein involved in epithelial–mesenchymal transition (EMT) [351].

### 6.6. EBV and circRNA

EBV encodes more than 30 different v-circRNAs from dissimilar regions of its genome, which are stably expressed in all EBV-associated tumors [352,353,366,367]. EBV-encoded circRNAs play a role in viral replication and facilitate EBV pathogenesis and tumor development. EBV circRNAs were shown to sponge host cell microRNAs such as miR-31, miR-203, and miR-451, promote proliferation, EMT and cell survival [351,353]. Other microRNA sequestered by EBV circRNAs allowed translation of their target mRNAs, resulting in increased protein levels of, e.g., E2F3, MAPK, checkpoint kinase 1 (CHEK1), and transforming growth factor beta 1 (TGFβ1). Enhanced expression of these proteins may contribute to EBV-induced carcinogenesis [313]. Some EBV circRNAs contain open reading frames and may encode putative peptides. One example is v-circBHLF1, which may be translated in a putative 200 amino acid peptide, but the existence of this protein remains to be confirmed [353].

### 6.7. KSHV and circRNA

KSHV produces more than 100 circRNAs, which can be detected in Kaposi’s sarcoma tumors, PEL, and multicentric Castleman’s disease [316,353,354,355]. The functions of v-circRNAs in KSHV’s pathogenesis are still largely enigmatic. Interestingly, the KSHV virion contain v-circRNAs, suggesting that they are important to establish infection and maybe exert a role as immune modulators [355]. Among virion-contained circRNAs is circ_0001400, which suppressed viral gene expression and thus may serve as an antiviral defense mechanism [354]. KSHV can also trigger the production of cellular circRNAs. Infection of endothelial cells with wild-type KSHV or KSHV with mutated vIRF1 or ectopic expression of vIRF1 demonstrated differential expression of several circRNAs. One of vIRF1-upregulated circRNAs was circARFGEF1. vIRF1 interacted with transcription factor lymphoid enhancer binding protein 1 (LEF1) and bound to the promoter region that produces the transcript from which circARFGEF1 is generated. The authors went on to show that circARFGEF1 could bind and degrade miR-125a-3p. Levels of glutaredoxin 3 (GLRX3), whose transcript is a miR-125a-3p target, were upregulated and knockdown of GLRX3 impaired motility, proliferation and angiogenesis. Accordingly, knockdown of circARFGEF1 or miR-125a-3p overexpression inhibited vIRF1-induced cell migration, proliferation and in vivo angiogenesis [356]. These results indicate that the vIRF1/circARFGEF1/miR-125a-3p/GLRX3 axis is essential for KSHV-induced invasion and angiogenesis.

### 6.8. HBV and circRNA

An HBV-encoded circRNA, HBV_circ_1, has been detected in HBV-infected cells and in HBV-associated HCC. HBV_circ_1 is mainly located in the cytoplasm and it was found to bind DXH9, as well as the ribosomal protein P0/P1/P2. Knockdown of DXH9 increased HBV_circ_1 levels which is in agreement with a previous study that described a role of DXH9 in repressing circRNA production [368]. Increased HBV_circ_1 levels or knockdown of DXH9 coincided with decreased levels of RNAs encoding the viral proteins. Hence, DXH9 may be an essential cellular factor in the regulation of HBV protein levels [357]. The mechanism by which HBV_circ_1 is produced and whether it may act as a decoy for microRNAs or other proteins remain to be elucidated.

There is compelling evidence that cellular circRNAs are involved in the etiology of HBV-associated HCC. By comparing the landscape of circRNA from HBV-positive HCC tissue and control tissue, differentially expressed cellular circRNAs were identified [351,358,369,370,371]. The role of some of these differentially expressed circRNAs in the pathogenesis of HBV was explored. For example, hsa_circRNA_100338 is upregulated and this circRNA acted as a sponge for miR-141-3p, a microRNA known to inhibit proliferation, migration and invasion and to regulate apoptosis [351]. Additionally, circ-RNF13 (=hsa_cric_0067717 or hsa_circ_103489) was upregulated in HBV-positive HCC tissue and cells compared with paired normal liver tissue or HBV-negative HCC cells. The authors showed that this circRNA sequestered miR-425-5p, which targets the TGFβ-induced homeobox 2 (TGIF2) transcript. Si-RNA mediated silencing of circ-RNF13 suppressed proliferation, migration, and invasion, and induced apoptosis in vitro, and suppressed tumor growth in vivo. Moreover, it blocked viral DNA replication and reduced the levels of hepatitis B surface and E antigens [358]. These examples show that HBV-induced circRNAs may play essential roles in HBV infection and HBV-positive HCC development.

## 7. Epigenetic Targeting Therapies for Treatment of Virus-Associated Tumors 

Oncovirus infection has a substantial impact on the host’s epigenetic landscape, which plays a crucial role in virus-driven oncogenesis. Reversing or preventing tumor virus induced epigenetic changes may therefore be a strategy for treating virus-associated tumors (for recent reviews [372,373,374]). A few examples will be discussed in this section.

As mentioned in Section 2, tumor viruses trigger often hypermethylation of tumor suppressor genes, resulting in silencing their expression. DNMT inhibitors can be used to reverse hypermethylation of these genes. The DNMT inhibitors 5-azacytidine and 5-aza-2′-deoxycytidine have been successfully used for treating patients with EBV-positive B cell lymphoma or HPV-positive cancers, respectively [375,376]. A recent study reported that infection of liver cells pretreated with 5-azacytidine with HBV and then challenged with IFNα, inhibited HBV replication by >50%, whereas no inhibition was measured in non-5-azacytidine treated cells [377]. This result illustrates that epigenetic reprogramming restores the antiviral activity of IFNα and suggests that demethylating drugs may have therapeutic potentials for treating HBV-infection and HBV-associated cancer.

In vitro and in vivo studies have demonstrated that HDAC inhibitors, such as the FDA approved drugs vorinostat, belinostat and panobinostat, could be a promising therapy for HPV-positive cancers [378]. A phase I/II study with the HDAC inhibitor entinostat is now recruiting patients with HPV associated malignancies (clinical trial study NCT04708470). Inhibitors of HAT are also being developed. One of them, the specific p300 inhibitor C646, reduces HR-HPV E6 and E7 expression in cervical cancer cells [379]. Several inhibitors against other histone modifying enzymes have been developed. One of the most studied is 3-deazaneplanocin (ZNep), which had a stronger anti-proliferative effect on HPV-positive oropharyngeal squamous cell carcinoma cell lines compared to virus-negative cell lines [380]. Inhibition of EZH2 with ZNep in HTLV-1 infected cells or ATL cells also reduced cell proliferation [74]. Inhibition of KDM1A with the drug GSK-LSD1 induced growth arrest and cell death of several MCPyV positive MCC cell lines and significantly reduced tumor growth in a xenograft model compared with vehicle treated animals. No synergistic effect was observed when HDAC and LSD1 inhibitors were used [381].

Anti-microRNAs have been designed to target specific microRNAs. The anti-miR-122 (Miravirsen) is used for treatment of HCV infections [382], and blocking of EBV microRNA BART17-5p, which targets the mRNA for tumor suppressor KLF2, suppressed the development of EBV associated gastric cancers [383].

How to exploit lncRNAs and circRNAs for therapeutic purposes in virus-associated cancers remains in its infancy. A recent study showed that a peptide that blocks the interaction between lncRNA HOTAIR and EZH2 decreased invasion of cancer cells in vitro and reduced tumor formation in ovarian tumor xenograft [384]. This may be relevant for virus-associated cancers because levels of HOTAIR are upregulated by several human tumor viruses (see Table 4). CRISPR/Cas9-mediated targeting of lncRNA UCA1 resulted in increased apoptosis and decreased cell proliferation, migration and invasion of bladder cancer cells in vitro and in vivo, but the application in virus-associated cancers expressing this lncRNA remains to be explored [385].

## 8. Conclusions

**Figure 1:**
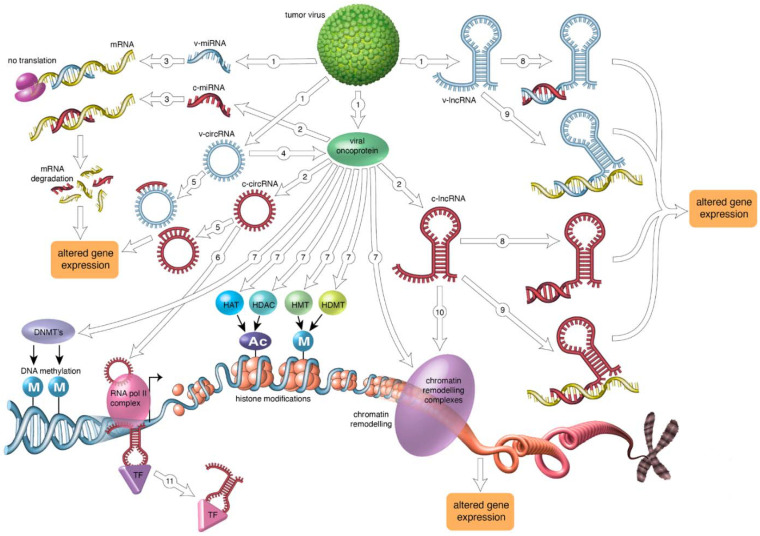
Epigenetic mechanisms by human tumor virus. (1) The virus encodes viral oncoproteins and its own v-microRNA, v-circRNA, and v-lncRNA. (2) Viral oncoproteins induce the expression of cellular microRNA (c-miRNA), c-circRNA, and c-lncRNA. (3) v-miRNA and c-miRNA can bind to target mRNA and induce mRNA degradation or prevent translation. (4) v-circRNA can be translated into a viral oncoprotein. (5) v-circRNA and c-circRNA act as a miRNA sponge. (6) c-circ interacts with the transcriptional machinery. (7) Viral oncoprotiens can regulate the expression of, can interact with, and can modulate the activity of DNA and histone modifying proteins and of components of chromatin remodeling complexes. (8) lncRNA sequesters miRNA. (9) lncRNA prevents translation of mRNA. (10) lncRNA recruits components of chromatin remodeling complexes. (11) lncRNA can modulate transcription by recruiting transcription factors (TF) or by sequestering TF to DNA.

All known human tumor viruses show great diversity in their structure and genome sequence. Their oncoproteins have no similarity, yet these viruses use the same mechanisms to induce cancer. They convey the hallmarks of cancer on the host cell. One way to obtain this is by altering gene expression in the infected cell and their viral proteins may do so by functioning as transcriptional regulators, by regulating the activity of transcriptional activators and repressors, or by inducing mutations in the host genome. During recent years, it has become clear that tumor viruses also apply epigenetic mechanisms to alter cellular gene expression. Again, all human tumor viruses seem to apply the same strategies (Figure 1). They can produce their own microRNA, lncRNA and circRNA or induce these cellular non-coding RNAs. Oncoviruses can modify DNA methylation, cause PTM on histones, and induce chromatin remodeling. However, several central questions remain to be elaborated. The mechanisms by which viruses affect these processes are incompletely characterized, and the biological implications of these epigenetic changes in virus-associated cancers are not always understood. As epigenetic changes progress over time [386], and many human tumor viruses have a long incubation, it is not always easy to attribute epigenetic modification to viral infection. Tumor virus infected cells can pack microRNAs, lncRNAs, and circRNAs into extracellular vesicles which can be taken up by other cells and RNA molecules can cause epigenetic changes in the recipient cell without viral infection. Tumor virus genomes may be lost after an epigenetic pattern has been established, supporting the hit-and-run hypothesis in tumor virology [387]. N^6^-methyladenosine RNA methylation adds another layer of complexity to epigenetic changes and has been shown to play a role in cancer [388]. Viral genomes and viral transcripts can be N^6^-methyladenosine modified and can have an effect on the viral life cycle and pathogenicity, as was shown for HCV and HBV [389]. N^6^-methyladenosine modification of circRNAs is not uncommon and plays a role in their regulation and function [390]. Two recent studies reported that the EBV infection induces changes in N^6^-methyladenosine RNA methylation of viral and host cell mRNA. These epitransciptomic changes promoted EBV infection in vitro [391,392]. Once more, viruses take advantages of cellular processes to favor their life cycle. Whether virus-mediated changes in N^6^-methyladenosine RNA methylation contributes to cancer remains unknown, but it would not be a surprise. Viruses keep amazing scientists with their creativity.

## Figures and Tables

**Table 1:** Effects of viral oncoproteins on DNA methylating/demethylating enzymes. See text for details.

Viral Oncoprotein	DNA Methylation/Demethylation Enzymes	References
HTLV-1		
Tax	MDB2 recruitment	[24,25]
Unknown	Increased DNMT1 and DNMT3B levels	[26]
HCV		
Core protein	Increased DNMT1 and DNMT3B levels	[27,28,29,30]
MCPyV	Unknown	
HR-HPV		
E6	Increased DNMT1 level	[31,32]
E7	Increased DNMT1 level and activity	[31,32]
EBV		
LMP1	Increased DNMT1, DNMT3A, and DNMT3B levels and activity	[33,34,35,36]
LMP1	Increased recruitment of DNMT1 to promoters	[37]
LMP1	Decreased DNMT1 level	[38]
LMP2A	Increased DNMT1 and DNMT3A levels	[39,40]
LMP2A	Decreased TET1 and TET2 levels	[41]
EBNA3C	Increased DNMT3A level	[42]
Unknown	Increased DNMT3A level	[38]
Unknown	Decreased DNMT3B level	[38]
KSHV		
LANA	Increased recruitment of DNMT3A to promoters	[43,44,45]
LANA	Increased recruitment of MeCP2 to promoters	[46]
vIRF1	Increased DNMT1 level	[47,48]
vIL6	Increased DNMT1 level	[49]
HBV		
HBx	Releasing DNMT3A from promoters	[50]
HBx	Increased DNMT1 level and recruitment	[51]
HBx	Increased DNMT3A level and recruitment	[52,53,54,55,56]
HBx	Decreased DNMT3B level	[52,54,55,56]
HBx	Recruitment of MeCP2	[52]
HBx	Decreased DNMT3A level	[52]
HBx	Decreased DNMT3L level	[50]
HBx	Increased recruitment of MeCP2	[50]
HBx	Increased recruitment of MBD1	[54]

**Table 2:** Effects of viral oncoproteins on histone modifying enzymes and protein chromatin remodeling complexes. See text for details.

Viral Oncoprotein	Histone Modifying Enzyme and Chromatin Remodeling Protein	References
HTLV-1		
Tax	Recruitment of p300/CBP	[147,148]
Tax	Recruitment of HDAC1	[149]
Tax	Recruits SIRT1	[150]
Tax	Increased EZH2 level and interaction with EZH2	[151]
Tax	Recruitment of SWI/SNF components BRG1, BAF53, BAF57, BAF155	[152]
Tax	Recruitment of CARM1/PRMT4	[75]
HBZ	Sequestering of p300/CBP	[147]
HBZ	Inhibition of KAT7 activity	[153]
HBZ	Impaired recruitment of KMT1A	[154]
HBZ	Displacement of BRF1	[152]
Unknown	Increased levels of the PRC2 complex proteins	[151]
Unknown	Recruitment of CTCF	[155]
HCV		
Core protein	Increased HDAC1 level	[156]
Core protein	Degradation of the PRC1 component RNF2	[157]
Unknown	Inactivation of PRMT1	[158]
MCPyV		
sT	Recruitment EP400 HAT and chromatin remodeling complex	[159]
sT	Increased expression *KDMA1* gene	[159]
HR-HPV		
E6	Inhibition of p300/CBP activity	[160]
E6	Stimulation proteasomal degradation of TIP60	[161]
E6	Increased EZH2 level	[162]
E6	Stimulation proteasomal degradation of KDM5C	[163]
E6	Inhibition of CARM1/PRMT4 activity	[164]
E6	Inhibition of PRMT1 activity	[164]
E6	Inhibition of KMT5A	[164]
E6/E7	Decreased level of the PRC1 protein BMI1	[165]
E7	Stimulation of p300/CBP activity	[166]
E7	Inhibition of PCAF/KAT2B activity	[167]
E7	Sequestering the NuR complex components Mi2β, HDAC1, and HDAC2	[168]
E7	Increased activity of BRG1	[169]
E7	Increased EZH2 level	[162]
E7	Increased KDM6A and KDM6B levels	[170]
Unknown	Increased HDAC1 and HDAC2 levels	[171]
EBV		
LMP1	Increased KDM6 levels	[172]
EBNA2	Stimulation of p300/CBP and PCAF/KAT2B activities	[173]
EBNA2	Recruitment of the chromatin remodeling complex INO80	[174]
EBNA3C	Inhibition of p300/CBP activity	[175]
EBNA3C	Recruitment HDAC1 and HDAC2	[176]
EBNA-LP	Recruitment of the chromatin remodeling complex INO80	[174]
BZLF1	Recruitment of p300/CBP	[177]
BZLF1	Recruitment of chromatin remodeling proteins SNF2h and INO80	[178]
BRLF1	Recruitment of p300/CBP	[179]
Unknown	Increased level of the SNF2 member LSH	[180]
KSHV		
LANA	SAP30	[181]
LANA	KMT1A/SUV39H1	[182]
LANA	Increased EZH2 level	[183]
LANA	Recruitment of KMT2F	[184]
LANA	Recruitment of BRD/BET	[185]
vIRF	Inhibits p300/CBP activity	[186]
vIRF3	Prevents nuclear export HDAC5	[187]
Rta	Recruitment of p300/CBP	[188]
Rta	Recruitment of BRG1	[188]
vIL6	Increased EZH2 level	[183]
vFLIP	Increased EZH2 level	[183]
HBV		
HBx	Recruitment of p300/CBP	[189,190]
	Recruitment of HDAC1	[50]
	Increased HDAC1, HDAC2, and HDAC3 levels and activities	[191,192]
	Increased SETDB1 level	[193]
	Increased EZH2 level	[56,194,195]
	Increased SMYD3 level	[196]
	Increased PRC2 activity	[192]
	Increased KDM1A activity	[192]
	Complex formation with RelA, EZH2, TET2, and DNMT3L	[197]
	Stabilization of WDR5 and recruitment to chromatin	[198]

**Table 3:** Some of the microRNAs affected by human tumor viruses. See text for details.

Virus	miR	Viral Protein	Expression	Target	Effect	References
HTLV-1	miR-150	Tax, HBZ	Down	STAT1	↑ proliferation; evade immune clearance	[235,236]
	miR-223	Tax, HBZ	Down	STAT1	↑ proliferation; evade immune clearance	[235,236]
	miR-17	HBZ	Up	HSSB2	↑ proliferation; genome instability	[237]
	miR-21	HBZ	Up	HSSB2	↑ proliferation; genome instability	[237]
	miR-23b	HBZ	Up	HSSB2	↑ proliferation; genome instability	[237]
	miR-27b	HBZ	Up	HSSB2	↑ proliferation; genome instability	[237]
HCV	miR-30c	Core	Down	SNAI1	↑ EMT; ↓ apoptosis	[238]
	miR-122	Core	Down	HCV DNA	↑ viral replication	[238]
	miR-124	Core	Down	SMYD3, EZH2	↑ migration and invasion	[238,239]
				TERT	Histone modification; ↑ proliferation	
	miR-138	Core	Down	WNT1	↑ immortality	[238]
	miR-152	Core	Down	SNAI2	↑ proliferation	[238]
	miR-203	Core	Down	PTEN	↑ EMT; ↓ apoptosis	[238]
	miR-21	Core	Up	IFNAR1	↑ proliferation; ↑ invasion	[238]
	miR-93	Core	Up	MCL1	evade antiviral effect	[238]
	miR-193b	Core	Up	FAS, TERT	↓ apoptosis	[238]
	miR-196b	Core	Up	TLR3, TLR7	↓ apoptosis; ↑ proliferation	[238]
	miR-758	Core	Up	ND *	Immune evasion	[238]
MCPyV	miR-M1	ND *	Up	LT, SP100	Immune evasion	[240,241]
	miR-30a-3p	ND	Up	ATG7, SQSTM1	Suppression of autophagy	[242]
	miR-30a-5p	ND	Up	ATG7, SQSTM1	Suppression of autophagy	[242]
	miR-34a	ND	Up	ND	ND	[243]
	miR-375	ND	Up	ATG7, SQSTM1,	Suppression of autophagy;	[242]
				LDHB	↑ proliferation	[244]
HR-HPV	miR-21	E6, E7	Up	CCL20	↑ proliferation; ↑ migration; ↓ apoptosis	[108]
	miR34a	E6	Down	cyclinD, BCL2	↑ proliferation; ↓ apoptosis	[108,245]
	miR-107	ND	Down	MCL1	Evade antiviral effect	[114]
	miR-124	ND	Down	IGFBP7	↑ proliferation; ↑ migration	[246]
	miR-155	ND	Up	LKB1	↑ proliferation	[245]
EBV	BART2-5p	ND	Up	MCIB	Immune evasion	[247,248,249]
	BART5-5p	ND	Up	PUMA	↓ apoptosis	[247,248,249]
	BART9	ND	Up	BIM	↓ apoptosis	[247,248,249]
	BART11	ND	Up	BIM	↓ apoptosis	[247,248,249]
	BART11	ND	Up	BIM	↓ apoptosis	[247,248,249]
	BART15	ND	Up	NLRP3	↑ inflammation	[247,248,249]
	miR-146-5p	EBNA2	Up	KDM2	Histone modification	[222]
	miR-155	LMP		FOXO3a	↑ proliferation	[248]
KSHV	miR-K12-1	ND	Up	p21^CIP1/WAF1^	↑ proliferation	[250,251,252]
	miR-K10	ND	Up	BCLAF1	↓ apoptosis	[250,251,252]
	miR-K12-5	ND	Up	BCLAF1	↓ apoptosis	[250,251,252]
	miR-K12-9	ND	Up	BCLAF1	↓ apoptosis	[250,251,252]
	miR-K12-1	ND	Up	Caspase 3	↓ apoptosis	[250,251,252]
	miR-K12-3	ND	Up	Caspase 3	↓ apoptosis	[250,251,252]
	miR-K12-4-3	ND	Up	Caspase 3	↓ apoptosis	[250,251,252]
	miR-K12-4-5	ND	Up	Rbl2	↓ DNA methylation	[253]
	miR-K12-11	ND	Up	Jarid	Histone modification	[253]
	miR-21	K15	Up	ND	↑ migration; ↑ angiogenesis	[248,252]
	miR-31	K15	Up	ND	↑ migration; ↑ angiogenesis	[248,252]
	miR-221	LANA, kaposin	Down	ETS2	↑ migration	[248,252]
		LANA, kaposin				
	miR-222	vFLIP	Down	ETS1	↑ migration	[248,252]
		ND				
	miR-146a		Up	CXCR4	↑ release KSHV-infected endothelial cells	[248,252]
	miR-132		Up	p300	↓ antiviral immunity	[254]
HBV	HBV-miR-2	ND	Up	TRIM35, RAN	↑ migration and invasion	[255]
	HBV-miR-3	ND	Up	PP1A, PTEN	↑ proliferation; ↑ invasion	[256]
	miR-10	HBx	Up	EPHA4	↑ EMT	[257]
	miR-132	HBx	Down	AKT	↑ proliferation	[257]
	miR-143	HBx	Up	FNDC3B	↑ migration, evasion, and metastasis	[257]
	miR-193b	HBx	Up	MCL1	Evade antiviral effect	[257]

↑ = increased; ↓ = decreased; * ND: not determined.

**Table 4:** Human tumor virus and lncRNAs with their targets and known functions in virus-induced cancer. See text for details.

Virus	lncRNA	Expression	Viral Protein	Target	Effect	References
HTLV-1	HBZ antisense	Up	ND *	*CCR4*, *E2F1*, and *survivin* genes	↑ proliferation; ↓ apoptosis	[302]
	ANRIL	Up	Tax, HBZ	Recruits EZH2 and p65	↑ proliferation; ↓ apoptosis	[303]
HCV	UCA1	Up	ND	miR-203 sponge → increased SNAI2	↑ EMT	[304]
	PVT1	Up	ND	Recruits EZH2	↑ proliferation	[304,305]
	AK021443	Up	ND	ND	↑ proliferation	[304,305]
	LINC01419	Up	ND	ND	↑ proliferation	[304,305]
	HULC	Up	ND	ND	↑ proliferation	[304,305]
	AF070632	Down	ND	ND	metabolism	[304,305]
MCPyV	HNGA1	Up	ND	miR-375 sponge	↑ proliferation; ↑ glycolysis	[306]
HR-HPV	HOTAIR	Up	ND	miR-23b and miR-143-3p sponge;	↑ angiogenesis; ↑ invasion	[245]
				Recruits EZH2; increased expression VEGF and MMP9		
	TMPOP2	Up	E6	miR-139 and miR-375 sponge → ↑ E6 and ↑ E7; inhibition of E-cadherin	↑ proliferation; ↑ invasion; ↑ angiogenesis	[307]
	CCDST	Down	E6, E7	Increased DHX9 level	↑ mobility; ↑ angiogenesis	[308]
	FANCI-2	Up	E6, E7	ND	ND	[309]
EBV	EBER1	Up	ND	ND	Immune evasion; ↓ apoptosis	[45,310,311]
	EBER2	Up	ND	Recruits PAX5	↓ apoptosis	[45,310,311]
	BART	Up	ND	↓ IFNB1 and ↓ CXCL8	Immune evasion	[312,313,314,315]
	BHLF1	Up	ND	Viral replication and latency	Persistent infection	[312,313,314,315]
	MALAT1	Up	ND	miR-124 and miR-195 sponge	↑ proliferation; ↑ invasion	[313]
	HOTAIR	Up	ND	miR-34a, miR-217 and miR-618 sponge	↑ angiogenesis; ↑ invasion and migration	[314]
	H19	Up	ND	miR-141 and miR-630 sponge	↓ apoptosis; ↑ invasion and migration	[314]
KSHV	PAN	Up	ND	Binds IRF4, histone demethylases, EZH2, and SUZ12	Immune evasion; chromatin modification	[316]
	H19	Up	ND	miR23b, miR-34a, miR124 sponge	Tumor progression and metastasis	[314]
	MALAT1	Up	ND	miR-124 and miR-195 sponge	↑ proliferation; ↑ invasion	[314]
	KIKAT	Up	ND	Interaction with KDM4A; ↑ ATOM	↑ angiogenesis; ↑ migration	[317]
HBV	HEIH	Up	HBx	Recruits EZH2	↑ proliferation; ↑ invasion; ↓ apoptosis	[318,319,320]
	UCA1	Up	ND	Recruits EZH2; miR-216b sponge	↑ proliferation; ↑ invasion; ↓ apoptosis	[318,319,320,321,322,323,324]
	HOTAIR	Up	ND	Recruits EZH2	↑ proliferation; ↑ invasion; ↓ apoptosis	[318,319,320,321]
	HULC	Up	HBx	Recruits EZH2; miR-186 and miR-372 sponge	↑ proliferation; ↑ invasion; ↓ apoptosis	[318,319,320,321,322]
	LINC00152	Up	HBx	Recruits EZH2	↑ proliferation; ↑ invasion; ↓ apoptosis	[319,325]
	ANRIL	Up	ND	Recruits PRC2; miR-122-5p sponge	↑ proliferation; ↑ invasion; ↓ apoptosis	[319]
	HBx-LINE1	Up	ND	Activates WNT pathway	↑ proliferation; ↑ invasion	[318,319,321,326]

↑ = increased; ↓ = decreased; * ND: not determined.

**Table 5:** Human tumor virus and circular RNAs with their targets and known functions in virus-induced oncogenesis. See text for details.

Virus	circRNA	Expression	Target	Effect	References
HTLV-1	ND	ND *	ND	ND	
HCV	circPSD3	Up	ND	↓ viral infectivity	[345]
MCPyV	circALTO1/2	Up	Sponge miR-M1; activation of some promoters; encodes ALTO protein	↑ LT expression	[346]
	circMCV-T		Sponge miR-M1	↑ LT expression	[347]
HR-HPV	circE7	Up	Encodes E7; sponge for several miRs	↑ proliferation; ↑ invasion; ↓ apoptosis; ↑ angiogenesis	[348,349]
	circRNA8924	Up	miR-518-d-5p and miR-519-59 sponge	↑ proliferation; ↑ invasion	[350]
	circ_0005576	Up	miR-153-3p sponge	↑ proliferation; ↑ invasion	[350]
	circ_0018239	Up	ND	↑ proliferation; ↑ invasion; ↑ immune evasion	[351]
	circ_0000263	Up	*TP53* mRNA	↑ proliferation; ↑ invasion; ↑ immune evasion	[351]
	circ_000284	Up	*SNAI2* mRNA	↑ proliferation; ↑ invasion; ↑ immune evasion	[351]
EBV	>30 EBV circRNAs	Up	miR-31, miR-203, and miR-451 sponge	↑ proliferation; ↑ EMT; ↓ apoptosis	[352,353]
	EBV circBHLF1	Up	Putative 200 aa protein	ND	[353]
KSHV	>100 KSHV circRNAs	Up	ND	Viral infection; immune modulation	[353,354,355]
	circ_0001400	Up	ND	Suppression viral expression	[354]
	circARFGEF1	Up	Sequesters miR-125a-3p → ↑ GLRX3	↑ proliferation; ↑ invasion; ↑ angiogenesis	[356]
HBV	HBV_circ_1	Up	Binds DXH9 and P0/P1/P2	Viral gene expression	[357]
	circ_100338	Up	miR-141-3p sponge	↑ proliferation; ↑ invasion; ↓ apoptosis	[351]
	circ-RNF13	Up	Sequesters miR-425-5p → ↑ TGIF2	↑ proliferation; ↑ invasion; ↓ apoptosis	[358]

↑ = increased; ↓ = decreased; * ND: not determined.

## Data Availability

Not applicable.

## Abbreviations

aHIF	antisense to hypoxia-inducible factor 1 alpha
AICDA	activation-induced cytidine deaminase
ALTO	alternative T open reading frame
ANRIL	antisense noncoding RNA in the INK4 locus
ATG7	autophagy related 7
ATL	adult T-cell leukemia-lymphoma
ATOH1	Atonal BHLH transcription factor 1
APC	adenomatous polyposis coli protein
ATOM	angiomotin
BAF	BRG-associated factor
BART	BHRF1 cluster and the BamHI-A rightward transcript
BCL2L11	BCL2 like 11
BCLAF	Bcl-2-associated factor 1
BHLF1	BamHI leftward reading frame 1
BIM	BCL2 interacting mediator of cell death
BMI1	B lymphoma murine leukemia virus insertion region 1
BRD4	bromodomain containing 4
BRG1	BRM/SWI2-related gene
CARM1	coactivator associated arginine methyltransferase 1
Cas9	CRISPR associated protein 9
CBP	CREB-binding protein
CCDST	cervical cancer DHX9 suppressive transcript
CCL20	C-C motif chemokine ligand 20
CCND2	cyclin D2
CCR4	C-C chemokine receptor type 4
CDH1	cadherin 1
CDKN1A	cyclin-dependent kinase inhibitor 1A or p21^CIP1/WAF1^
CDKN2A	cyclin-dependent kinase inhibitor 2A or p14^ARF^
CDKN2B	cyclin-dependent kinase inhibitor 2B or p14^INK4B^
CENP-B	centromere protein B
c-FOS	FBJ murine osteosarcoma viral oncogene homolog
CHD	chromodomain helicase DNA-binding protein
CHEK1	checkpoint kinase 1
circRNA	circular RNA
c-MYC	avian myelocytomatosis viral oncogene homolog
CRE	cAMP-response element
CREB	CRE binding protein
CRISPR	clustered regularly interspaced short palindromic sequences
CTCF	CCCTC-binding factor
CXCL	C-X-C motif chemokine ligand
CXCR	C-X-C motif chemokine receptor
DHX9	DExH-box helicase 9
DLEU2	deleted in lymphocytic leukemia 2
DNMT	DNA methyltransferase
DREH	downregulated expression by HBx
DUSP2	dual specificity phosphatase 2
EBER	EBV-encoded RNA
EBV	Epstein-Barr virus
EED	embryonic ectoderm development
EGF	epidermal growth factor
EGR3	early growth response 3
EMT	epithelial-mesenchymal transition
EpCAM	epithelial cell adhesion molecule
EPHA4	ephrin receptor 4
ETS	E-twenty six transcription factor
EVC	Ellis Van Creveld
EZH	enhancer of zeste homolog
FANCI-2	Fanconi anemia complementation group 1-2
FCER2	Fc fragment of IgE receptor II
FHIT	fragile histidine triad diadenosine triphosphate
FNDC3B	fibronectin type III domain containing 3B
FOX	Forkhead box
GAS5	growth arrest specific 5
GLRX3	glutaredoxin 3
GLUT1	glucose transporter type 1
GSTP1	glutathione S-transferase Pi 1
H3K4me3	trimethylation of histone 3 at lysine 4
H3K9ac	acetylation of histone 3 at lysine 9
H3K9me3	trimethylation of histone 3 at lysine 9
H3K27me3	trimethylation of histone 3 at lysine 27
H4K16ac	acetylation of histone 4 at lysine 16
H4R3me	methylation of histone 4 at arginine 3
H4K20me	methylation of histone 4 at lysine 20
HAT	histone acetyltransferase
HBV	hepatitis B virus
HBZ	basic zipper
HCC	hepatocellular carcinoma
HCV	hepatitis C virus
HDAC	histone deacetylases
HEIH	highly expressed in HCC
HHV4	human herpes virus-4
HHV8	human herpesvirus-8
HIF-1α	hypoxia-inducible factor 1 alpha
HLTF	helicase like transcription factor
HNGA1	head and neck squamous cell carcinoma glycolysis-associated 1
HOTAIR	HOX antisense intergenic RNA
HOX	homeobox
HR-HPV	high-risk human papillomavirus
HTLV-1	human T-lymphotropic virus 1
HULC	highly upregulated in liver cancer
IFNAR1	interferon α and β receptor subunit 1
IFNB1	interferon beta 1
IGFBP	insulin like growth factor binding protein
IL-8	interleukin 8
INO80	inositol requiring 80
IRAK1	interleukin-1 receptor-associated kinase 1
IRF4	interferon regulatory factor 4
ISWI	imitation switch
JNK	c-Jun N-terminal kinase
K	lysine
KAT2B	lysine acetyltransferase 2B
KAT7	lysine acetyltransferase 7 (or HBO1: histone acetyltransferase binding to ORC1)
KDM2B	lysine-specific demethylase 2B
KIKAT	KSHV-induced KDM4A-associated transcript
KLF4	Kruppel-like factor
KMT	lysine methyltransferase
KSHV	Kaposi’s sarcoma-associated herpes virus
LANA	latency-associated nuclear antigen
LDHB	lactate dehydrogenase B
LEF1	lymphoid enhancer binding protein 1
LINE1	long interspersed nuclear element 1
LKB1	liver kinase B1
lncRNA	long non-coding RNA
LSH	lymphoid-specific helicase
LT	large tumor antigen
LTR	long terminal repeat
MALAT1	metastasis-associated lung adenocarcinoma transcript 1 (=NEAT2)
M6A	N6-methyladenosine
MAPK	mitogen-activated protein kinase
MBD	methyl-CpG-binding domain
MCC	Merkel cell carcinoma
MCIB	MHC class I polypeptide-related sequence B
MCL1	myeloid cell leukemia sequence 1
MCPyV	Merkel cell polyomavirus
MeCP2	methyl CpG binding protein 2
MHC-I	major histocompatibility complex class I
MMP9	matrix metalloproteinase 9
MYD88	myeloid differentiation primary response 88
NABP1	nucleic acid binding protein 1
NDRG2	N-myc downregulated gene 2
NEAT2	nuclear-enriched abundant transcript 2
NFκB	nuclear factor kappa B
NLRP3	NLR family pyrin domain containing 3 or cryopin
NuRD	nucleosome remodeling complex
PAN	polyadenylated nuclear RNA
PBMC	peripheral blood mononuclear cells
PCAF	p300/CBP-associated factor
PCNA	proliferating cell nuclear antigen
PDCD	programmed cell death
PEL	primary effusion lymphoma
PI3K	phosphatidylinositol 3-kinase
PLK1	polo like kinase 1
PP1R13B	protein phosphatase 1 regulatory subunit 13B
PPP2CA	protein phosphatase 2 catalytic subunit alpha
PRC	polycomb repressive complex
PRDM	PR/SET domain
PRLH1	p53-regulated lncRNA for homologous recombination repair 1
PRMT	protein arginine methyltransferase
PSD	pleckstrin and sec 7 domain containing
PTCH1	Patched 1
PTEN	phosphatase and tensin homolog
PTM	posttranslational modification
PTPRG	protein tyrosine phosphatase receptor type G
PUMA	p53 upregulated modulator of apoptosis
PVT1	plasmacytoma variant translocation 1
R	arginine
RASSF	Ras associated domain family member
RB1	retinoblastoma 1 or pRB
RbAp	retinoblastoma-binding protein
RBL2	retinoblastoma transcriptional corepressor like 2
REST	repressor element 1 silencing transcription factor
RIG-1	retinoic acid-inducible gene 1 protein
RISC	RNA-inducing silencing complex
RNF	ring finger protein 2
S	serine
SAF	Fas-Antisense
SAP30	Sin3-associated protein 30
SCL2A1	solute carrier family 2 member 1
SETD1A	SET domain containing 1A
SFRP1	secreted frizzled related protein 1
SH3BGR	SH3 domain binding glutamate-rich protein
SHP-1	Src homology-2-containing protein tyrosine phosphatase 1
SIN3A	switch independent 3A
SIRT1	sirtuin 1
SMYD3	set and mynd domain containing
SNAI	snail family transcriptional repressor
SOCS1	suppressor of cytokine signaling 1
SQSTM1	sequestosome 1
sT	small tumor antigen
STAT	Signal transducer and activator of transcription
SUV39H1	suppressor of variegation 3-9 homolog 1 (=KMT1A)
SUZ12	suppressor of zeste 12 homolog
SWI/SNF	switching defective/sucrose non-fermentable
SYK	spleen associated tyrosine kinase
T	threonine
TDG	thymine DNA glycosylase
TET	ten-eleven translocation
TERT	telomerase reverse transcriptase
TGFβ1	transforming growth factor beta 1
TGFBR2	tumor growth factor-beta type II receptor
TGIF2	TGFβ-induced homeobox 2
TLR	toll-like receptor
TMPOP2	thymopoietin pseudogene 2
TP53BP2	tumor promoter p53 binding protein 2
TP73	tumor protein p73
TRIM35	tripartite motif containing 35
TUSC7	tumor suppressor candidate 7
UCA1	urothelial carcinoma associated 1
UHRF	ubiquitin-like, containing PHD and RING finger domain
VCAM	vascular cell adhesion molecule 1
VEGF	vascular endothelial growth factor
vFLIP	viral FLICE inhibitory protein
vIL-6	viral interleukin 6
WDR5	WD repeat domain 5
WNT1	Wnt family member 1
Y	tyrosine
ZBTB	Zinc finger and BTB domain containing
